# Antimicrobial peptides modulate lung injury by altering the intestinal microbiota

**DOI:** 10.1186/s40168-023-01673-0

**Published:** 2023-10-16

**Authors:** Ahmed Abdelgawad, Teodora Nicola, Isaac Martin, Brian A. Halloran, Kosuke Tanaka, Comfort Y. Adegboye, Pankaj Jain, Changchun Ren, Charitharth V. Lal, Namasivayam Ambalavanan, Amy E. O’Connell, Tamás Jilling, Kent A. Willis

**Affiliations:** 1 Division of Neonatology, Department of Pediatrics, Heersink School of Medicine, University of Alabama at Birmingham, Birmingham, AL USA; 2 Division of Newborn Medicine, Department of Pediatrics, Boston Children’s Hospital, Harvard Medical School, Boston, MA USA

**Keywords:** Microbiome, Gut-lung axis, Chronic lung disease, Lysozyme, Neonatal lung injury, Neonate, Bronchopulmonary dysplasia, Post-prematurity lung disease

## Abstract

**Background:**

Mammalian mucosal barriers secrete antimicrobial peptides (AMPs) as critical, host-derived regulators of the microbiota. However, mechanisms that support microbiota homeostasis in response to inflammatory stimuli, such as supraphysiologic oxygen, remain unclear.

**Results:**

We show that supraphysiologic oxygen exposure to neonatal mice, or direct exposure of intestinal organoids to supraphysiologic oxygen, suppresses the intestinal expression of AMPs and alters intestinal microbiota composition. Oral supplementation of the prototypical AMP lysozyme to hyperoxia-exposed neonatal mice reduced hyperoxia-induced alterations in their microbiota and was associated with decreased lung injury.

**Conclusions:**

Our results identify a gut-lung axis driven by intestinal AMP expression and mediated by the intestinal microbiota that is linked to lung injury in newborns. Together, these data support that intestinal AMPs modulate lung injury and repair.

Video Abstract

**Supplementary Information:**

The online version contains supplementary material available at 10.1186/s40168-023-01673-0.

## Background

Fetal lung development is a tightly regulated process at low oxygen tension [1]. However, premature birth exposes the developing lung to far greater oxygen concentrations, which is usually compounded by the clinical use of supplemental oxygen and mechanical ventilation after birth [2]. The direct effects of supraphysiologic oxygen on the lungs are well described [3], but there is also evidence for concurrent systemic, non-pulmonary oxygen toxicity exemplified by retinopathy of prematurity [3]. However, the effect of supraphysiologic oxygen on the host-microbiota interface of the neonatal intestine remains unclear.

Antimicrobial peptides (AMPs) are an extensive class of proteins secreted across mucosal surfaces that have central roles in responding to inflammation and regulating the commensal microbiota [4]. In addition to antimicrobial properties, AMPs have anti-inflammatory, wound-healing, and tissue-protective effects [5]. In the newborn, intestinal and nasopharyngeal secretion of AMPs increases with age, potentially a necessary suppression to aid the establishment of a normal microbiota or to compensate for the intake of milk-derived AMPs [4]. Since AMP expression also increases in response to inflammation [5], supraphysiologic oxygen might disrupt initial microbial colonization.

While exploring the effects of hyperoxia exposure on the transcriptional landscape of the developing gut and lungs, we were intrigued by alterations in AMP production in the neonatal intestine associated with microbiota alterations. Here, we asked if hyperoxia-induced alterations in intestinal AMP expression could alter the composition of the intestinal microbiota, creating a feedback loop that modulates inflammation in the developing lung.

## Methods

### Experimental model details

#### Animals

Animal experimentation was performed at the University of Alabama at Birmingham under protocol IRB140926096. To broaden the translatability and reproducibility of the resulting experiments, we intentionally selected two similar, but not identical, mouse strains that we purchased from different animal vendors. We raised the mice in different vivaria to further vary their initial microbiomes. In the first experiment, we used 8-week-old female C57Bl/6 J mice (Stock Number 000664) purchased from the Jackson Laboratory (hence, JAX). We allowed them to acclimate in specific-pathogen-free (SPF) housing with 4 animals per cage on a 12-h light/dark cycle, at 20-24C, with ad libitum access to irradiated rodent chow (Teklad 7904, Envigo) in SPF Vivarium 1 (Pittman Biomedical Research Building II) for two weeks. Similarly, for the second experiment, we purchased C57Bl/6NCrl mice (Stock Number 027) from the Charles River Laboratory and acclimated them in SPF Vivarium 2 (Volker Hall) with the same diet for two weeks. Animal studies were performed in accordance with the recommendations in the Guide for the Care and Use of Laboratory Animals of the National Institutes of Health, under protocol 22042, approved by the Institutional Animal Care and Use Committee at UAB. Animal experimentation is reported in accordance with the ARRIVE guidelines [6]. We utilized our established, standardized hyperoxia-exposure model of BPD [7, 8]. We time-mated the mice to produce multiple simultaneous birth cohorts with the same perinatal exposure. On the day of birth, pups from two consecutively born litters with the same perinatal exposure were pooled, and the pups were re-distributed evenly between the two dams. Litter sizes for all experiments were adjusted to 5–8 pups per treatment group to minimize nutritional effects on lung development, and the sex of the pups was evenly distributed between the two litters. One pooled litter was randomly assigned to hyperoxia [fraction of inspired oxygen (FiO_2_) 0.85]) and the other to air (FiO_2_ 0.21). Pups were exposed continuously for 11 days, from the third day after birth until the 14th day (P3-14). Oxygen concentrations were maintained using a ProOx monitor (Biospherix). Dams were rotated daily between the two pooled litters to limit maternal complications of hyperoxia. Growth was monitored daily for all pups throughout the experiment.

For the lysozyme exposure experiment, pups from two pooled litters at a time were randomized on P2 to either lysozyme exposure or vehicle exposure. Lysozyme exposure was performed via every other day oral gavage of 10,000 units/gram in PBS to pups marked by tail clipping. Unmarked littermates were used as a vehicle (PBS)–exposed controls. Experiments were repeated at least three times.

### Mouse intestinal epithelial spheroid organoids

Organoid modeling was performed at Boston Children’s Hospital. Crypts were collected from C57Bl6J mice, cultured in 50 mL Matrigel, and fed with 500 mL murine enteroid media in a 24-well plate in a 37 °C humidified incubator with 5% CO_2_. Media was changed every 2–3 days for the duration of the cultures. Organoids were passaged every 7–10 days by dissolving the Matrigel in Cell Recovery Media with mechanical disruption.

### Method details

#### Forced oscillometry

A subset of P14 mice not used for morphometry or transcriptomics were sedated with ketamine and xylazine via I.P. injection, and the flexiVent system (SCIREQ) was used to assess respiratory resistance and compliance as described [9, 10]. The mice were tracheotomized with the appropriately sized cannula secured with a silk ligature. The flexiVent then executed the neonatal mouse pulmonary function program using room air in the sedated, closed-chest animal. Calibration of the flexiVent was done using the tracheal cannula before each experiment. Lung volumes were measured by volume displacement after completion of the flexiVent measurements.

#### Histology and morphometry

The right lung was gravity inflated to 20 cmH_2_O via tracheal insertion of an angiocath with 4% formalin as described [7]. Three random 4–5 µm sections from each lung were stained with hematoxylin and eosin. To perform morphometry, lung sections were digitized under × 20 magnification and analyzed independently by two researchers blinded to the group assignments using established methods [7, 11–13]. Alveolarization was quantified using the mean linear intercept (*L*_*m*_) and radial alveolar counts (RAC).

#### Generation of intestinal organoids

Organoids were generated from C57Bl/6 mice after euthanasia using CO_2,_ followed by cervical dislocation. Proximal duodenal tissue was dissected, and feculent matter was cleared using cold intraluminal PBS. The tissue was cut longitudinally, then cut into 0.5 cm pieces, and cleaned repeatedly in PBS until the supernatant was cleared. The tissue was then placed in 2 mM EDTA and incubated on ice with rocking for 30 min. The tissue was then shaken vigorously for 2 min to release crypts and pipetted up and down 25 times with a 10 mL serological pipet while mixing simultaneously. The solution was passed through a 70uM filter, and the filtrate was collected into a new 50 mL conical tube. The filtrate was centrifuged (5 min at 300xg), and the supernatant was discarded. Dissociated cells were then washed three times in Advanced DMEM/F12, centrifuged (5 min at 300xg) after each wash, and reconstituted in Matrigel (50uL/well) and plated in 24-well plates and incubated for 10 min at 37 C. Next, 0.5 mL growth media was added to the wells. Enteroids were fed with growth media every 2–3 days and passaged approximately every 7 days.

#### Organoid hyperoxia experiment

Intestinal organoids were generated and incubated in a hyperoxia chamber (StemCell Technologies) at 95% oxygen for 24 h. After the hyperoxia was completed, the supernatant was removed, and the organoids and Matrigel were collected in 0.5 mL cell recovery media (Corning) per well and placed on ice for 40 min. The solution was then centrifuged (5 min at 300 g at 4C), and the supernatant was removed. The organoids were then either placed in 2% b-mercaptoethanol in RLT buffer or 4% paraformaldehyde for RNA isolation or fixation, respectively. For each experimental condition, 100 organoids were evaluated per experimental well. It was determined whether the epithelial rim was clear, indicating a healthy cellular environment and an intact organoid, or whether the epithelium was dark. The epithelial cells were damaged, indicating a loss of organoid integrity suggestive of necrosis. The number displayed is the number of organoids with damaged cells per 100 organoids counted, and the results are displayed as a percentage.

#### Immunohistochemistry

Organoids were kept in 1.5 ml Eppendorf tubes. Three tubes per group were fixed by 0.4% PFA. Samples were resuspended using a Wide Bore Pipet Tips in a blocking buffer (1% BSA, 1% normal donkey serum, 1% CD16/32 in PBS) for 30 min in RT and then incubated for one hour with primary antibodies at RT in incubation buffer (1% BSA, 1% normal donkey serum, 0.3% Triton X-100, and 0.01% sodium azide in PBS). The samples were then washed in PBS and incubated for one hour with the corresponding secondary antibodies at RT in the incubation buffer. Samples were centrifuged and resuspended in the proper buffer for each step. Finally, samples were counterstained with Click-iT™ EdU Cell Proliferation Kit for Imaging, Alexa Fluor™ 488. Images were then captured using an A1R HD inverted confocal microscope.

#### Transcriptomics

25 mg tissue sections from either the lung's left lower lobe or the terminal ileum were bead-lysed in RLT buffer (Qiagen) supplemented with 1% (w/w) 2-mercaptoethanol and frozen. RNA was isolated from cells with an RNeasy kit with Qiashredder cell disruption (Qiagen) and RNase-free DNase Set (Qiagen) on-column DNA digestion. DNA purity and concentration were assessed using an Agilent 2100 Bioanalyzer RNA Analysis chip (Eukaryote Total RNA Pico Series II) (Agilent Technologies). Samples from Exp. 1 underwent transcriptomic analysis using array-based gene expression analysis, and samples from Exp. 2 using RNAseq.

#### For array-based gene expression analysis

Gene expression analysis was performed using the MouseWG-6 v2.0 array (Illumina), following quality testing of mRNA using an Agilent 2100 Bioanalyzer. Data were analyzed in GeneSpring GX using two-way ANOVA, and the FDR threshold (q) < 0.05 was calculated according to the Benjamini–Hochberg method. A fold change cutoff of ≥  ± 2 generated downstream data sets.

#### For RNAseq

Preparation of RNA library (mRNA library preparation (poly A enrichment) and transcriptome sequencing (NovaSeq PE150 (6 G raw data per sample) was conducted by Novogene Co., LTD, using the HiSeq 2500 platform (Illumina) with a pair-end 150-nucleotide read length as described [14]. Raw sequencing data was mapped to the genome (GRCm39, mRNA GRCm39.109), raw and normalized counts (TPM) were determined, and differential gene expression was calculated using the CLC Genomic Workbench (GUI version, Qiagen). Genes with adjusted *p*-value (q) < 0.05 and log_10_(FoldChange) > 0.301 were considered as differentially expressed. Heatmaps and PC analyses/plots were generated using *ClustVis* [15]. Differentially expressed genes were input for Ingenuity IPA (Qiagen) to identify significantly regulated networks and pathways. Differentially regulated genes were sometimes used as input for Gene Set Enrichment Analysis (v 3.3.2; Broad Institute and UC San Diego). A gene set enrichment map was generated using Cytoscape.

#### Microbiome analysis

A 1 cm section of liquid nitrogen snap-frozen terminal ileum was used to quantify the mouse intestinal microbiome because of the advantages in quantifying the adherent mucosal microbiota [16]. We used ZymoBIOMICS whole organism and DNA microbial community standards (Zymo Research) and sterile-filtered PBS to create pairs of positive and negative controls appropriate for each step of the sample collection and isolation procedure. Microbial DNA was extracted and sequenced using the MiSeq platform (Illumina) at the UAB Microbiome Core using 16S V3-4 (bacterial) primers 515F-806R. We performed Sybr Green RT qPCR for universal 16S primers of samples and both positive and negative controls with the following conditions: 96 C 5 min, cycle: 96 C 10 s, 53 C 10 s, and 68 C 20 s.

#### Bioinformatics

We used QIIME 2.0 to merge, cluster, and quality control 16S rRNA gene amplicon sequences. Operational taxonomic units (OTUs) were called based on the SILVA database using closed reference-based OTU clustering with PyNAST and taxonomy via UCLUST. OTU tables were exported into R, where they were decontaminated using *microDecon* and analyzed with the following packages: *vegan*, *tidyverse, phyloseq*, *DESeq2, Maaslin2*, *SpiecEasi, caret, psych, and ggcorrplot*. Two samples were excluded for insufficient read depth (below 1000 OTUs). The Shannon and Simpson Indices quantified alpha diversity using the raw OTU data. Significance testing was performed using Mann–Whitney U. We described beta diversity with principal coordinates analysis of Bray–Curtis distances and performed significance testing using PERMANOVA and PERMDISP. Additional analyses were performed using weighted UniFrac. Differential abundance analysis was performed with DESeq2 and MaAsLin2. Feature selection was performed with Random Forest, utilizing the *ranger* package within *caret*. Correlation analysis was performed between AMP qPCR data and the most significant OTUs from DESeq2 or random forest using the Spearman correlation.

#### Statistical analysis

Statistical analyses were performed in R, CLC Genomic Workbench or GraphPad Prism. In general, q < 0.05 after adjustment for multiple comparisons was considered statistically significant.

## Results

### Hyperoxia exposure alters lung morphology and function

**Fig. 1:**
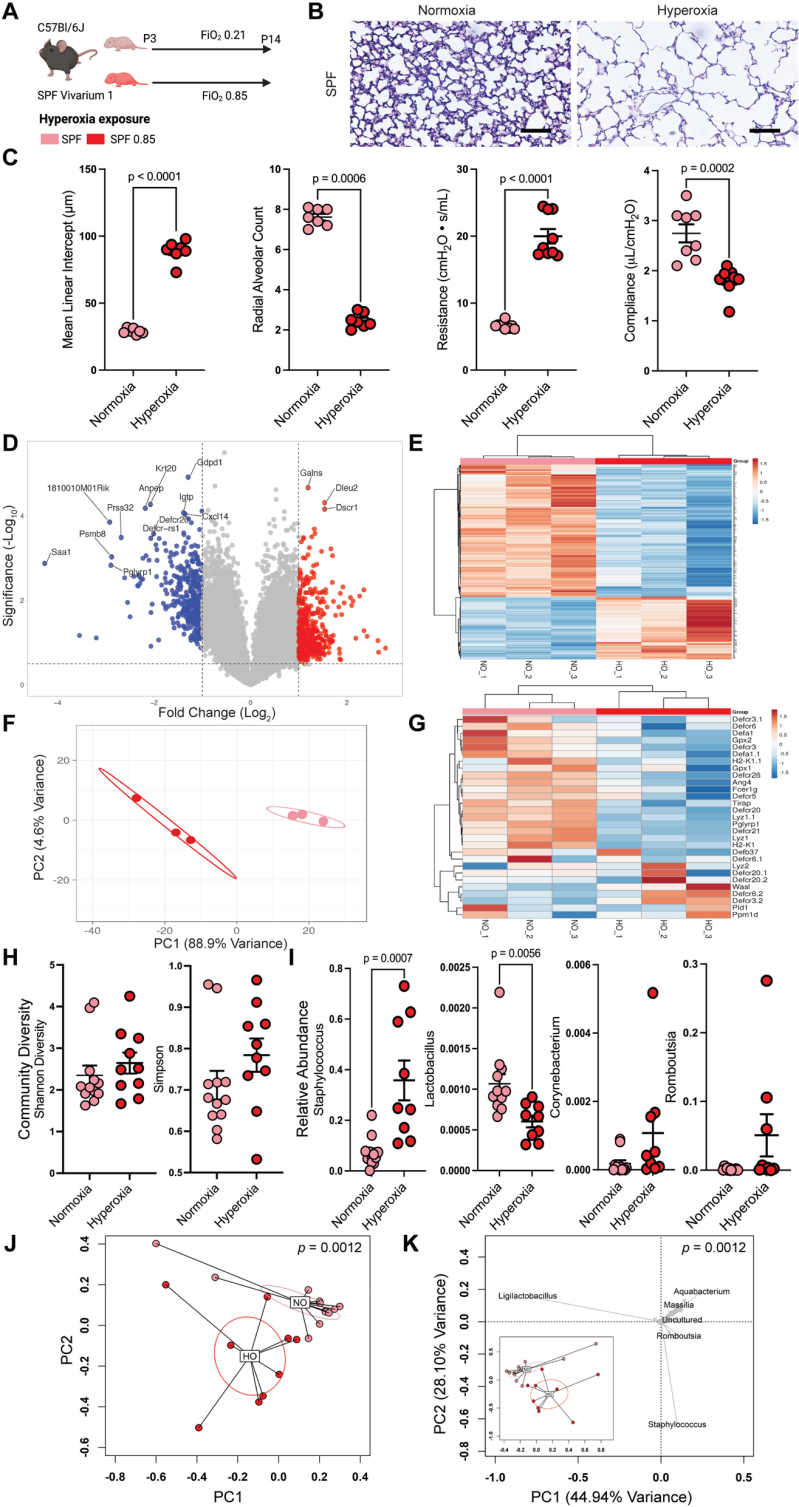
Oxygen exposure reduces intestinal antimicrobial peptide expression. **A** Neonatal C57BL/6 J mice were exposed to normoxia or hyperoxia from the 3^rd^-14.^th^ day of life (*n* = 4 litters with 5–7 neonatal mice/litter per exposure group). FiO_2_, fraction of inspired oxygen. SPF, specific-pathogen-free. **B** Representative photomicrographs of the distal lung sections of 14-day-old mice. **C** Hyperoxia exposure is associated with alterations in lung morphology and function. Data are shown as mean ± SEM, with significance testing by a two-tailed *t*-test. **D** Volcano plot of ileal gene expression array showing gene expression altered by hyperoxia exposure. **E** Heatmap showing genes regulated by hyperoxia exposure. **F** Principal components analysis showing differential clustering of normoxia and hyperoxia exposed ileal genes. PC, principal component. **G** Ileal antimicrobial peptide expression is decreased in hyperoxia-exposure mice. **H** Community diversity of the adherent and luminal ileal bacterial microbiome is not significantly altered by hyperoxia exposure. **I** The relative abundance of an operational taxonomic unit (OTU 002) that aligns to the genus *Staphylococcus* increases after hyperoxia exposure, as do OTUs aligning to *Corynebacterium* (OTU 124) and *Romboutsia* (OTU 013). Data are shown as mean ± SEM, with significance testing by a two-tailed *t*-test. **J** Principal coordinates analysis of Bray–Curtis dissimilarity shows global alterations in community composition in hyperoxia-exposed mice. Significance testing by permutational ANOVA (PERMANOVA), with equivocal dispersion confirmed by permutational multivariate analysis of dispersion (PERMDISP). PC, principal component. **K** Loading plot of principal components analysis of Hellinger transformed Euclidian distances showing the contribution of specific genera to the global community composition. Schematic in (**A**) was generated using BioRender. See also Figures S1, S2 and S5

To examine potential mechanistic connections between the effects of supraphysiological oxygen exposure on the intestine and on the lungs, we utilized a well-established model of bronchopulmonary dysplasia [7, 8] and exposed newborn mouse pups, pooled from simultaneously born litters and redistributed evenly by sex and number between the dams, to hyperoxia (fraction of inspired oxygen, FiO_2_, 0.85), and their littermate controls to normoxia (FiO_2_ 0.21), from the third to the 14th day of life (P3-P14, Fig. 1A). We reproduced well-described hyperoxia-induced alterations in lung structure and function (Fig. 1B, C) [17]. We also observed changes in the pulmonary transcriptome and related regulatory pathways (Fig. S1) that are consistent with similar observations [9, 18–20] showing type 2 immune polarization and extensive alterations in alveolar epithelial, endothelial, and fibroblast cell populations in response to hyperoxia-induced lung injury, such as the well-described increase in IL-6 and TNF-a (*IL6*: 158 fold, *q* < 1.87 × 10^–8^, *TNFA*: 3.39 fold, *q* = 0.01).

### Intestinal antimicrobial peptide expression is altered in hyperoxia-exposed neonatal mice

To interrogate potential hyperoxia-induced alterations in the neonatal gut, we then examined transcriptomic changes in the terminal ileum, where preterm infants are uniquely vulnerable to the microbiome-mediated disease necrotizing enterocolitis. Intriguingly, we found that hyperoxia-exposed mice had a significantly altered ileal transcriptome (Fig. 1D-G). Global gene expression patterns were significantly altered (Fig. 1D-F), including the prototypical AMP lysozyme (*Lyz1*, 4.2-fold decrease, *q* = 0.012, Fig. 1G, Figure S4, Table S1). AMPS were a key significantly regulated pathway by IPA (Figure S4). In addition to other AMPs, additional markers of ileal Paneth cells were also decreased (*Pnliprp2*, 5.0-fold decrease; *Reg3g*, 2.7-fold decrease; and *Guca2b*, 2.4-fold decrease. Table S1). *Tnfa*, *Il6,* and *Il1b* were not significantly increased in response to hyperoxia in intestinal tissue. We verified these alterations in AMPs using qPCR (Figure S5A). Together, these results suggest that hyperoxia exposure reduces the expression of intestinal AMPs.

### The intestinal microbiota responds to pulmonary hyperoxia exposure

We have reported that axenic (microbiota-free) mice devoid of a microbiome are protected [10], while antibiotic-exposed mice with a disrupted microbiome are more susceptible to hyperoxia-induced lung injury [7]. Together, these and other studies [11], suggest that the intestinal microbiota may modulate hyperoxia-induced lung injury. Because of this evidence and due to our novel observation of changes in the transcription of potentially microbiota-altering AMPs, we asked if hyperoxia exposure altered the intestinal microbiota. We specifically chose to focus on the terminal ileum because this is a uniquely active area of host-microbe interactions. To answer this question, we collected the intact terminal ileum from hyperoxia-exposed neonatal mice and littermate controls, which had been raised under specific pathogen-free (SPF) conditions, and performed 16S rRNA MiSeq to define both the adherent and luminal ileal microbiome.

We found that the terminal ileum has bacterial colonization, approximately a third of the density of stool in newborn mice (4 million versus 12 million copies of the 16S rRNA gene/mg. Figure S1). After exacting computational sequencing decontamination (Figure S2), we found the global community composition of the ileal microbiota was significantly altered by exposure to hyperoxia (*p* = 0.0012, *pseudo-F* = 5.2924, permutational ANOVA (PERMANOVA) of Bray–Curtis dissimilarity, *p* = 0.207, permutational multivariate analysis of dispersion (PERMDISP), but not diversity, not the alpha diversity (Fig. 1H-K, S5A, B). The principal components analysis's first and second principal components are driven primarily by the abundance of *Staphylococcus* and several uncultured *Ligilactobacillus* species (Fig. 1J). In unadjusted univariate analysis, we identified increases in taxa aligning to the genera *Staphylococcus* and *Lactobacillus* (Fig. 1I). We verified these alterations using negative binomial regression (Figure S5C), which also detected significant alterations in *Romboutsia, Proteus, Corynebacterium, Bacteroides*, *Herbaspirillum,* and other uncultured species. We also used random forest machine learning to identify *Staphylococcus*, *Ligilactobaccilus,* and *Bacillus* as potential important genera (Figure S5D). Correlation analysis between these differentially abundant genera and our validation qPCR for key AMPs supports a direct link between the expression of *Lyz1* and the relative abundance of *Staphylococcus* (Figure S5F). Together, these results suggest hyperoxia exposure alters the ileal microbiome by lysozyme decreasing expression, which was associated with increased relative abundance of *Staphylococcus*.

### Hyperoxia directly suppresses intestinal epithelial AMP expression

**Fig. 2:**
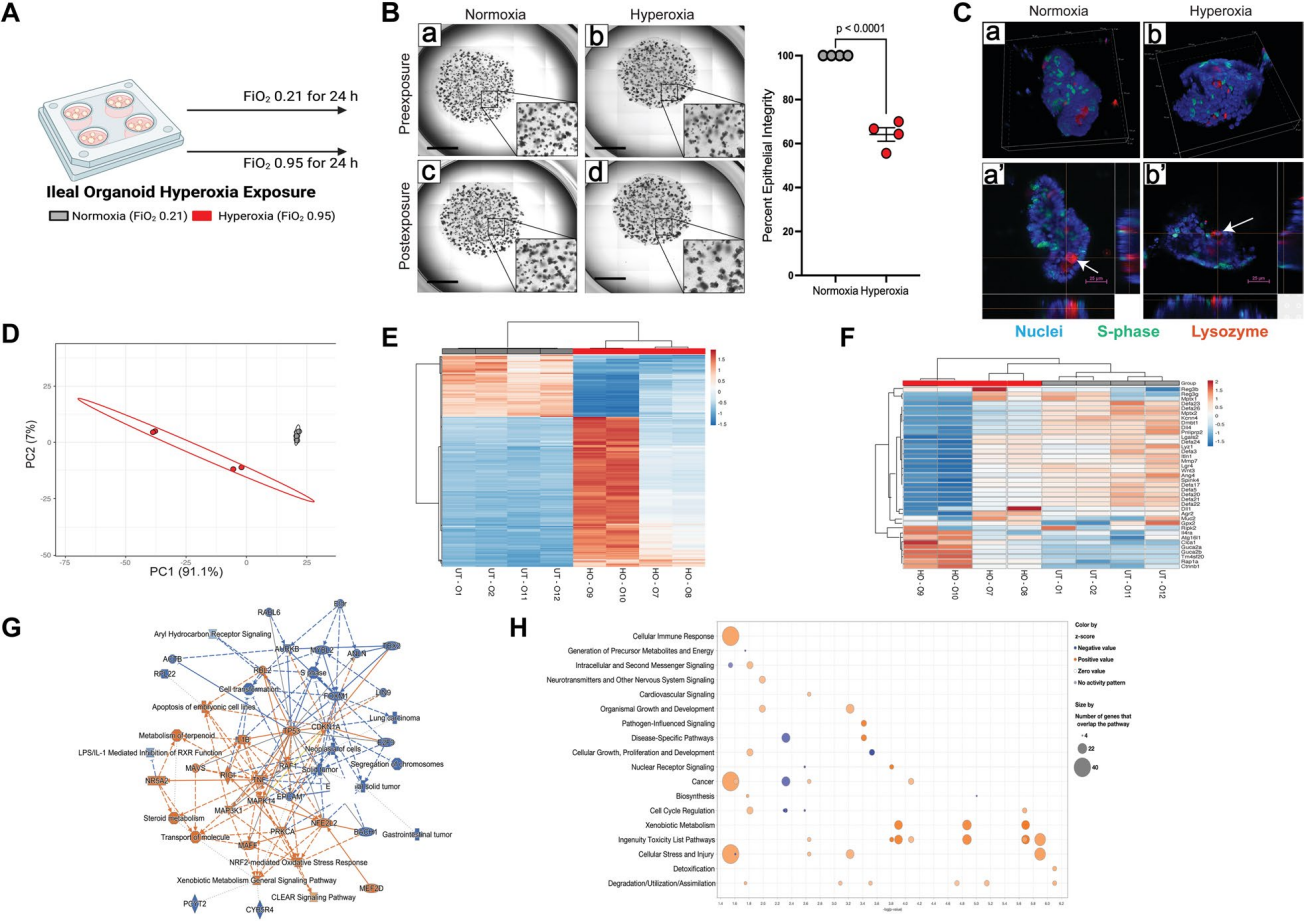
Antimicrobial peptide expression is reduced in hyperoxia-exposed intestinal organoids. **A** Small intestinal spheroid organoids derived from neonatal C57BL/6 J mice were exposed to either hyperoxia or normoxia for 24 h (*n* = 6 wells/treatment group). **B** Representative images of organoids before and after exposure, with insets at 40 × magnification. The percentage of organoids with healthy-appearing epithelium declined in hyperoxia-exposed organoids. Data are shown as mean ± SEM, with significance testing by a two-tailed *t*-test. The scale bar represents 1000 mm. **C** Representative immunohistochemistry after exposure to normoxia or hyperoxia. Nuclei in blue, actively proliferating cells in green, and lysozyme-positive cells in red. Arrows identify lysozyme-positive Paneth cells. The scale bar represents 25 mm. **D** Principal components analysis showing differential clustering of normoxia and hyperoxia exposed ileal genes. PC, principal component. **E** Heatmap showing genes regulated by hyperoxia exposure. **F** Heatmap of antimicrobial peptide expression is decreased in hyperoxia-exposure organoids. **G** Ingenuity pathway analysis showing regulated pathways in hyperoxia or normoxia. **H** Bubble plot showing up and down-regulated pathways from hyperoxia exposure. The schematic in (**A**) was generated using BioRender

We turned to small intestinal organoids to directly test the capacity for hyperoxia exposure to depress intestinal AMP expression. We exposed organoids to either hyperoxia (FiO_2_ 0.95) or normoxia (FiO_2_ 0.21) for 24 h (Fig. 2A). In general, live imaging with visual light microscopy and immunohistochemistry demonstrated organoids maintained similar shape and cellular composition in both normoxia and hyperoxia (Fig. 2B, C, Figure S6), but with a decrease in epithelial integrity (*p* < 0.0001, two-tailed *t*-test, Fig. 2B). RNAseq revealed transcriptomic changes that generally recapitulated the changes observed in hyperoxia-exposed mice (Fig. 2D-G). Principal components analysis shows a clear separation in gene expression between hyperoxia and normoxia-exposed organoids (Fig. 2D), which is also reflected in a heatmap of regulated genes (Fig. 2E). Pathway analysis showed extensive upregulation of genes associated with cellular inflammatory response and response to injury ( Fig. 2G). *Tnfa* increased slightly (1.59 fold, *q* = 0.04), but not *Il6* or *Il1b.* Like in vivo, we also noted a decrease in several Paneth cell markers (Table S2). We verified the relative expression of several genes by qPCR (Figure S5). As indicated in our transcriptomic data, *Lyz1* was decreased in mice exposed to hyperoxia, as was the defensin *Defa2* and the Paneth cell marker *Pnliprp2* (Figure S5A). Together, these data suggest that the downregulation of AMP expression is linked to cellular damage response of the ileal epithelium from exposure to supraphysiologic oxygen.

### Augmentation of lysozyme improves lung function in hyperoxia-exposed mice

**Fig. 3:**
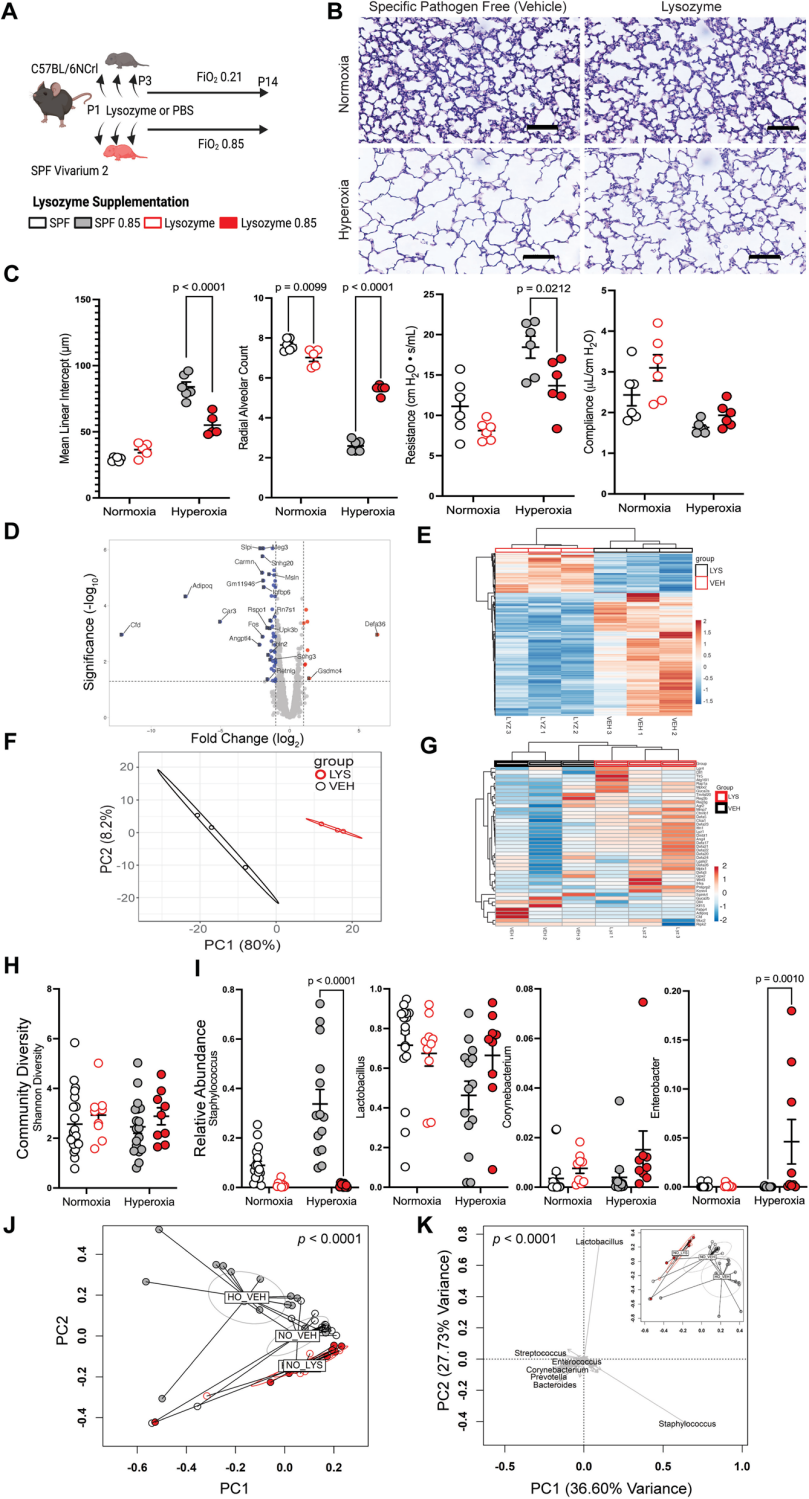
Intestinal lysozyme supplementation reduces hyperoxia-induced lung injury. **A** Neonatal C57BL/6NCrl mice randomized to either every other day exposure to lysozyme by gastric gavage or their littermate controls were then exposed to normoxia or hyperoxia from the 3^rd^-14.^th^ day of life (*n* = 4 litters with 5–7 neonatal mice/litter per exposure group). FiO_2_, fraction of inspired oxygen. PBS, phosphate-buffered saline (vehicle). SPF, specific-pathogen-free. **B** Representative photomicrographs of the distal lung sections of 14-day-old mice. **C** Lysozyme exposure ameliorates hyperoxia-induced disruptions in lung morphology and function. Data are shown as mean ± SEM, with significance testing by two-way ANOVA. **D** Volcano plot of ileal RNAseq showing gene expression altered by lysozyme exposure. **E** Heatmap showing genes regulated by lysozyme exposure. **F** Principal components analysis showing differential clustering of ileal genes in lysozyme-exposed mice. PC, principal component. **G** Ileal antimicrobial peptide expression is altered in lysozyme-exposed mice. **H** The community diversity of the adherent and luminal ileal bacterial microbiome is not significantly altered by lysozyme exposure. **I** The hyperoxia-induced increase in the relative abundance of operational taxonomic unit 014 (*Staphylococcus*) is ameliorated by lysozyme exposure. Multiple other genera are increased in lysozyme and hyperoxia-exposed mice. Data are shown as mean ± SEM, with significance testing by two-way ANOVA. **J** Principal coordinates analysis of Bray–Curtis dissimilarity shows global alterations in community composition in lysozyme-exposed mice. Significance testing by permutational ANOVA (PERMANOVA), with equivocal dispersion confirmed by permutational multivariate analysis of dispersion (PERMDISP). PC, principal component. **K** Loading plot of a principal components analysis of a Hellinger transformed Euclidian distance showing global community composition significantly altered in lysozyme-exposed mice. The schematic in (**A**) was generated using BioRender. See also Figure S7

Having shown that exposure to supraphysiologic oxygen resulted in depressed expression of intestinal AMPs and an altered microbiome, we next wanted to test if AMP expression drove this effect. Lysozyme is the principal AMP, and expression is reduced in early life on most mucosal surfaces [12]. To test if the suppression of AMP expression in hyperoxia-exposed mice might have functional consequences, we repeated the hyperoxia-exposure experiment above using nearly genetically identical mice (C57BL/6NCrl, Fig. 3A). However, to naturally vary their microbiota as much as possible, we purchased these animals from a different vendor and raised them in a different SPF vivarium. We exposed a randomly selected subset of their neonate to lysozyme by oral gavage and compared them to unexposed littermates. Surprisingly, augmenting intestinal lysozyme improved lung structure and function after hyperoxia exposure (MLI *p* < 0.0001, RAC *p* < 0.0001, and resistance *p* = 0.0212, two-way ANOVA, Fig. 3B, C).

We performed RNAseq using the terminal ileum of normoxia-exposed mice to identify alterations in intestinal AMP expression resulting from lysozyme exposure. Intriguingly, we found that lysozyme-exposed mice had a significantly altered ileal transcriptome (Fig. 3D-G). Global gene expression patterns were significantly altered (Fig. 3D-F), including altered expression of multiple AMPs (Fig. 3G). Of note, *Lyz1* expression increased 2.433-fold (FDR *p* = 0.0004). Together, these results suggest that dietary lysozyme exposure alters the expression of the ileal transcriptome and results in additional ileal lysozyme production in an apparent feed-forward mechanism.

### Lysozyme alters the intestinal microbiota

We next asked if supplementary lysozyme altered the intestinal microbiota. Oral lysozyme supplementation produced a marked change in the overall composition of the ileal microbiota as compared to littermate controls (*p* < 0.0001, pseudo*-F* = 5.8764, PERMANOVA; *p* = 0.7131, PERMDISP, Figs. 3J and S7), without significantly altering the community diversity (Fig. 3H and S7A). On univariate analysis, multiple species were differentially abundant (Fig. 3I). Most intriguing, the robust increase of *Staphylococcus* in hyperoxia-exposed Jackson Laboratory mice, was replicated in the vehicle and hyperoxia-exposed SPF Charles River mice but suppressed in lysozyme-exposed animals (*p* < 0.0001, two-way ANOVA, Fig. 3I). *Enterobacter* also increased in these doubly exposed mice (*p* = 0.0010, two-way ANOVA, Fig. 3H). MaAsLin 2 and binomial regression identified a shared 62 OTUs altered by lysozyme exposure (Figure S4). Together, these analyses suggest that augmenting dietary lysozyme results in a robust alteration of the ileal microbiome and ameliorates the increase in *Staphylococcus* that otherwise occurs with hyperoxia exposure.

### Lysozyme augmentation alters the pulmonary transcriptome

**Fig. 4:**
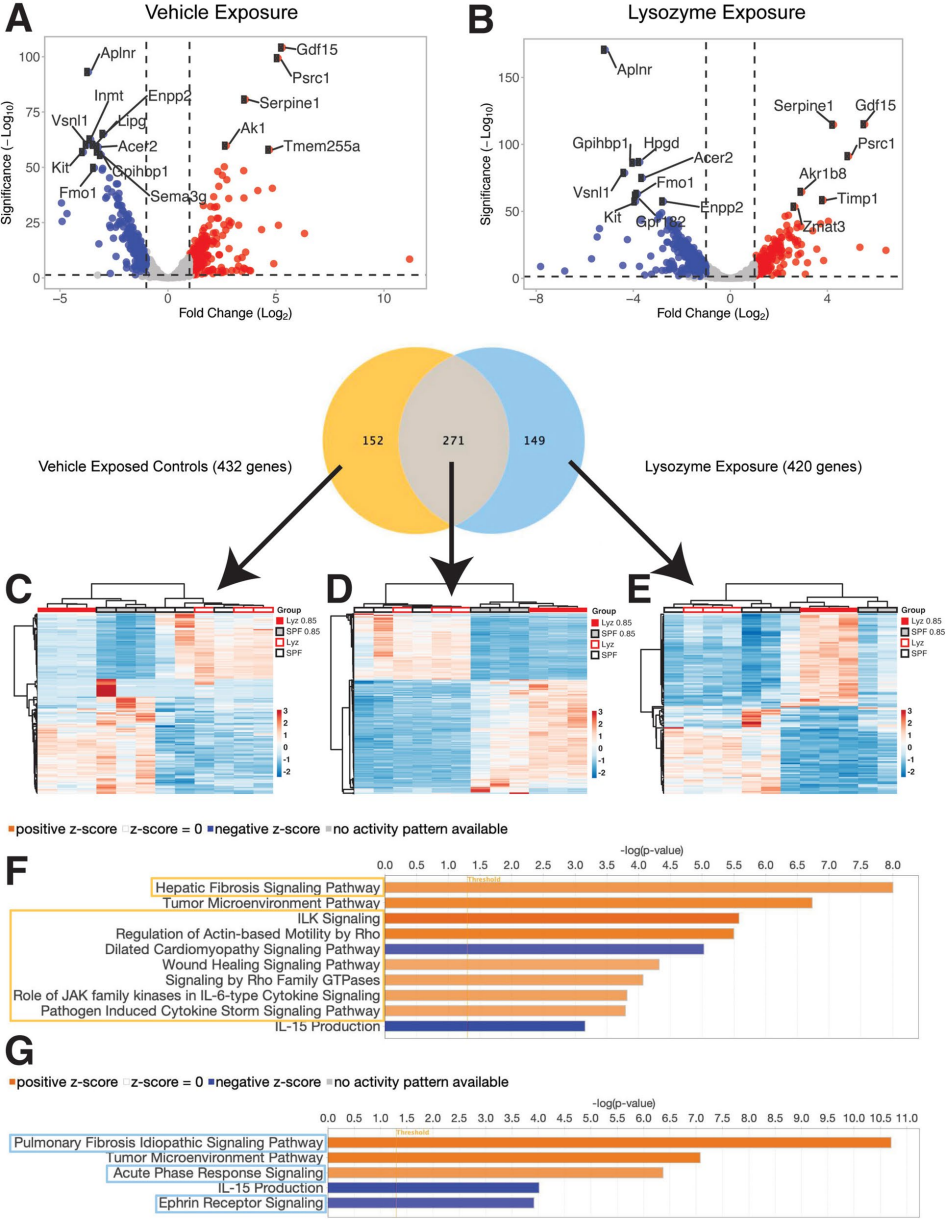
Lysozyme exposure alters the lung transcriptome. **A** Volcano plot showing hyperoxia alters gene expression in vehicle-exposed controls. **B** Volcano plot showing lysozyme exposure alters gene expression in the lung. **C** Heatmap showing differentially expressed genes in vehicle-exposed controls. **D** Heatmap showing similarly expressed genes between all groups. **E** Heatmap showing differentially expressed genes in lysozyme-exposed mice. **F** Major pathways altered in mice only exposed to normoxia or hyperoxia. **G** Major pathways altered in lysozyme-exposed mice

We next sought to examine if the functional improvements we identified in the lungs of mice exposed to lysozyme were associated with differences in the lung transcriptome. Indeed, RNAseq revealed extensive alterations in gene expression in the lung (Fig. 4). While the most increased inflammatory related genes remained elevated in lysozyme and hyperoxia-exposed mice they were reduced as compared to controls exposed only to hyperoxia (Table S3). Similar patterns exist for genes related to endothelial regulation (Table S4). However, multiple genes exhibited opposite regulation in lysozyme-exposed mice (Table S5). Overall, the most reduced genes in hyperoxia exposure, were reduced regardless of lysozyme exposure (Fig. 4A, B). The genes with the most increased expression were similar (Fig. 4A, B). However, notable differences in *Hpgd*, *Akr1b8*, *Timp1* and *Zmat3* were appreciable. Altogether, 432 genes were differentially regulated in vehicle-exposed controls (Fig. 4C) of which 271 were also differentially regulated the lysozyme exposed mice (Fig. 4D), leaving 149 unique differentially regulated genes in lysozyme exposed mice (Fig. 4E). The most significantly upregulated pathways in vehicle-exposed controls, included fibrosis, wound healing and IL-6-type cytokine signaling-related pathways (Fig. 4F). However, different fibrosis-related and acute phase response pathways were upregulated in lysozyme-exposed mice (Fig. 4F). Together, these transcriptional differences characterize a unique response to hyperoxia in the lungs of mice exposed to lysozyme, that is associated with ameliorated injury and an improvement in lung structure and function.

## Discussion

Here, we have shown that exposure of neonatal mice to supraphysiologic oxygen, in addition to the known effect of causing defective lung development, alters their intestinal AMP expression and their intestinal microbiome, and we provide evidence that the changes in intestinal microbiome contribute to lung injury. Hyperoxia inhibited AMP transcription, both in vivo in neonatal mice and in vitro in intestinal organoids. In vivo, this altered the intestinal microbiota. Conversely, augmenting intestinal AMP levels by oral administration of lysozyme altered the intestinal microbiota. It became more similar to room air-exposed mice and decreased the severity of their lung injury. This reciprocal relationship between intestinal AMPs, microbiome, and lung injury is consistent with the notion that a gut-lung axis mechanism can influence hyperoxia-exposure-induced lung injury. Together, these findings suggest intestinal AMPs are potential modulators of lung injury and repair due to their role in mediating microbiome composition.

AMPs are small cationic peptides that play a key role in host defense against microbes [13]. AMPs have broad antimicrobial activity and can kill or inhibit the growth of bacteria, fungi, and viruses [21]. AMPs, therefore, also play a key role in regulating intestinal microbiota [13]. However, the potential bioactive effects of AMPs are not exclusively related to manipulating the microbiota. Lysozyme, the major AMP, can directly reduce the propagation of reactive oxygen species in colitis by releasing bacterial superoxide dismutase from *Lactobacillus lactis* [22]. Numerous species of bacteria within the microbiota contain superoxide dismutase genes [22]. This suggests potential mechanisms by which alterations might influence the redox balance of the intestinal epithelium in AMP expression.

Multiple potential links between the lung and the gut have been proposed to explain the gut-lung axis [23]. Samuelson and colleagues [24] have proposed that microbes or microbial components may be trafficked to the lung via the lymph. Similarly, metabolites produced or modified by the intestinal microbiota, such as small-chain fatty acids, may also influence lung physiology [25]. Lysozyme can also liberate ligands for nucleotide-binding oligomerization domain containing 1 (Nod1) from bacterial cell wall peptidoglycans. Circulating Nod1 ligands may reach the lung directly or may participate in immune cell maturation in the gut to determine the set points of the innate immune system in early life [26, 27], and to educate the adaptive immune system [28]. While in this stage of our studies, we have not directly addressed these potential intermediate mechanisms, the AMP-mediated gut-lung axis we describe could impact some or all these potential pathways.

Exposure to supplemental oxygen is one of the most ubiquitous clinical interventions. Preterm infants, particularly the most immature ones, often require prolonged exposure [2]. The disastrous effects of such exposure on the structure and function of neonatal lungs are well described [3]. In the adult gut, the gradient between hypoxia and anoxia across the radial axis of the gut profoundly influences the composition of the intestinal microbiota. It maintains distinct communities of adherent and luminal microbes [29, 30]. Hyperbaric oxygen exposure can shift the composition of these mature communities [29, 30], as can inflammation, which alters the oxygen gradient in enterocytes [31]. However, the maturation of intestinal microbial communities is a developmental process, and initially, during the newborn period, the gut lumen is less hypoxic [32]. Recent evidence suggests that this process is driven by the accumulation of microbial biomass in the more distal intestine [32]. This is supported by the developmental progression from aerobic to facultative anaerobic microbes in the neonatal gut, and by the observation that while the intestinal lumen of axenic mice is anaerobic, this appears to be driven by a slower chemical process [32]. This suggests our findings may be influenced by a unique developmental window created by the immature gut being more vulnerable to oxidative stress and the immature microbiota less capable of functioning as an anoxic sink.

Lysozyme is a component of human milk with concentrations as high as ~ 1 g/L at some stages of lactation [33]. In a double-blind, randomized, placebo-controlled trial, supplementation of lysozyme and lactoferrin to children aged 12–23 months in rural Malawi resulted in improved gut health as determined by their ingested lactulose excreted into the urine, along with decreased rates of hospitalization [34]. Supplementation of lysozyme to the diet has been studied extensively in farm animal husbandry and has been documented to benefit intestinal development, function, and overall growth, and provides protection against pathogen challenge, mainly due to beneficial effects on the microbiome [35–38]. Therefore, assessing the supplementation of exogenous lysozyme to prevent abnormal pulmonary development in premature neonates may be worthy of further investigation.

In adult mice, hyperoxia exposure has been shown to alter the lung and intestinal microbiota. Ashley and colleagues observed an increase in the oxygen-tolerant genera *Staphylococcus* after 72 h of exposure to supraphysiological oxygen [11]. We observed a similar increase in *Staphylococcus* in the ileal microbiota of neonatal mice exposed to hyperoxia that replicated robustly in mice with different starting microbiomes. While Ashley and colleagues confirmed a previously described [31] decrease in the Ruminococcaceae after hyperoxia exposure in the cecal microbiota of these adult mice [11], several differences likely explain our divergent findings. First, we sampled the ileal microbiota, which is more translationally relevant to neonatal intestinal pathology. Second, our neonatal mice were considerably smaller and exposed to hyperoxia for longer, which would be intolerable for adult mice. Finally, their intestinal microbiota were in an earlier stage of community formation, which places their microbial communities under considerably different ecological pressures. Intriguingly, this increase in *Staphylococcus* was ameliorated in our lysozyme-exposed and associated with less severe lung injury. In subsequent experiments, Ashley and colleagues further found that adult axenic mice were protected from lung injury, and antibiotic-exposed mice experienced worse lung injury [11] —findings we have previously demonstrated in neonatal mice [7, 10].

Under SPF conditions, the intestinal microbiota of co-housed mice converges over time [39, 40], and the primary source of the intestinal microbiota is almost universally the mother but may show some littermate effects [41, 42]. We took multiple steps to address these factors in these experiments. First, we intentionally varied the initial microbiota of the maternal mice as much as possible by purchasing them from two different animal vendors, cage-randomizing them for cohousing on arrival, and acclimatizing them in two different SPF vivaria before breeding. Second, to limit a littermate effect, we took the newborn mice from two simultaneously born litters and randomly redistributed them between their two mothers on the day of birth. We then assigned one pooled litter to hyperoxia and the other to normoxia and rotated the mothers daily to minimize their unique contributions. For exposure to lysozyme, we randomly selected newborn mice within these homogenized litters and used their unexposed littermates as controls. Therefore, while this approach likely underestimates the potential microbiota effects of lysozyme exposure due to littermate cross-contamination, it underscores that the increase in *Staphylococcus* abundance with hyperoxia exposure is robust, since we replicated it across a wide range of initial microbiomes.

In summary, we have described a gut-lung axis driven by intestinal AMP expression and mediated by the intestinal microbiota that influences hyperoxia-induced lung injury. These murine and organoid experiments suggest that AMP expression represents a potential therapeutic target to modulate the intestinal microbiota and the response to lung injury. These results have implications for the clinical management of premature infants at high risk of developing bronchopulmonary dysplasia in neonatal care.

### Supplementary Information

**Additional file 1:** Key Resource Table. **Figure S1.** Ileal intestinal colonization is a lower biomass than stool by RT-qPCR. **Figure S2.** Quality control figure showing sequencing contaminates removed by microDecon. **Figure S3.** Hyperoxia exposure alters the mouse lung transcriptome. **Figure S4.** Hyperoxia exposure alters the mouse ileal transcriptome. Pathway analysis and GSEA corresponding to Figure 1 D-G. **Figure S5.** Hyperoxia alters the ileal microbiome. **Figure S6.** Hyperoxia exposure alters the morphology of intestinal organoids. **Figure S7.** Lysozyme and hyperoxia alter the ileal microbiome. **Table S1.** Paneth cell markers significantly changed in the murine ileum in response to hyperoxia. **Table S2.** Paneth cell markers significantly changed in the murine intestinal organoids in response to hyperoxia. **Table S3.** Lung inflammation-related genes as compared to normoxia vehicle exposed mice. **Table S4.** Genes-related to endothelial cell regulation. **Table S5.** Genes with opposite regulation associated with lysozyme supplementation. **Table S6.** Enrichment analysis table.

## Data Availability

16S and RNA sequencing data were deposited at the NCBI Sequence Read Archive (BioProject ID: PRJNA931604). Gene expression array data were deposited at the NCBI Gene Expression Omnibus (Accession number: GSE125489). Processed data files are available at http://github.com/WillisLungLab/AMP. This manuscript does not report original code. All other data needed to evaluate the conclusions in the manuscript are available within the main text or supplementary materials.
