# Supplementary material for: Antimicrobial peptides modulate lung injury by altering the intestinal microbiota
Source: Microbiome. 2023 Oct 16;11:226. doi: 10.1186/s40168-023-01673-0 (PMC10578018; doi:10.1186/s40168-023-01673-0)
Supplement: Supplementary file 2 — Additional file 1: Key Resource Table. Figure S1. Ileal intestinal colonization is a lower biomass than stool by RT-qPCR. Figure S2. Quality control figure showing sequencing contaminates removed by microDecon. Figure S3. Hyperoxia exposure alters the mouse lung transcriptome. Figure S4. Hyperoxia exposure alters the mouse ileal transcriptome. Pathway analysis and GSEA corresponding to Figure 1 D-G. Figure S5. Hyperoxia alters the ileal microbiome. Figure S6. Hyperoxia exposure alters the morphology of intestinal organoids. Figure S7. Lysozyme and hyperoxia alter the ileal microbiome. Table S1. Paneth cell markers significantly changed in the murine ileum in response to hyperoxia. Table S2. Paneth cell markers significantly changed in the murine intestinal organoids in response to hyperoxia. Table S3. Lung inflammation-related genes as compared to normoxia vehicle exposed mice. Table S4. Genes-related to endothelial cell regulation. Table S5. Genes with opposite regulation associated with lysozyme supplementation. Table S6. Enrichment analysis table. [file 40168_2023_1673_MOESM1_ESM.pdf]

Antimicrobial peptides modulate lung injury by altering the intestinal microbiota

Ahmed Abdelgawad*, Teodora Nicola*, Isaac Martin, Brian A. Halloran, Kosuke Tanaka, Comfort Y. Adegboye, Pankaj Jain, Changchun Ren, Charitharth V. Lal, Namasivayam Ambalavanan, Amy E. O'Connell[§], Tamás Jilling[§] and Kent A. Willis[§]

*Contributed equally
[§]Contributed equally

ONLINE DATA SUPPLEMENT

Graphical Abstract:

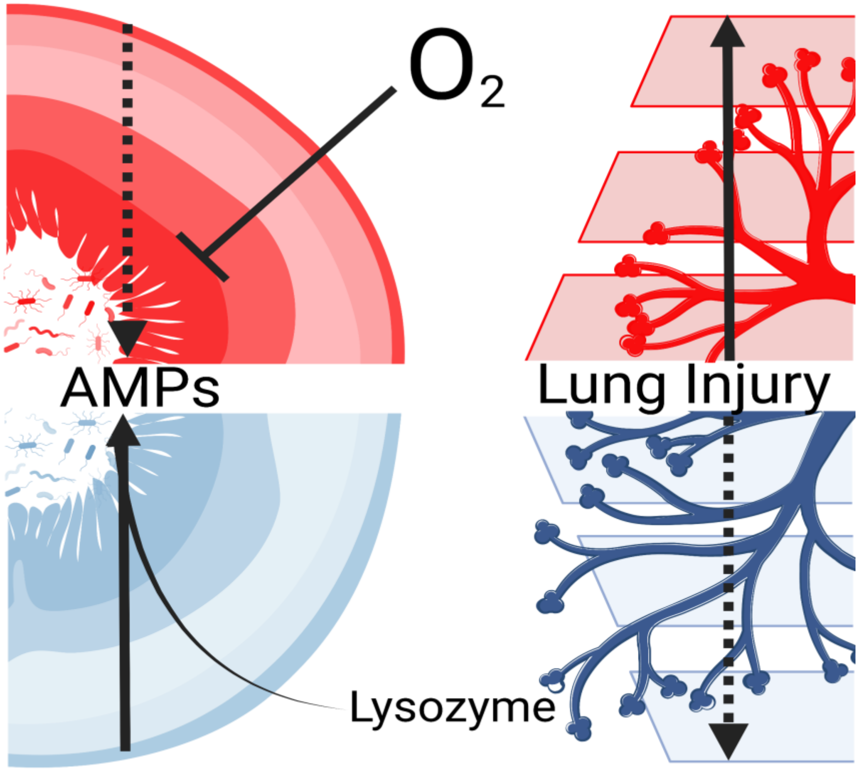

Key Resource Table

**Reagent**	**Company - Catalog #**	**RRID**
Teklad S-2335 Mouse Breeder Sterilizable Diet	Envigo - 7904.15	
Lysozyme	Sigma Aldrich 12671-19-1	
zymoBIOMICS microbial community standards	Zymo Research - D6300 and D6310	
zymoBIOMICS microbial community DNA standards	Zymo Research - D6305/6306 and D6311	
zymoBIOMICS spike-in control I	Zymo Research - D6320-10	
zymoBIOMICS spike-in control II	Zymo Research - D6321-10	
zymoBIOMICS gut microbiome standard	Zymo Research - D6331	
RNase-free DNase Set	Qiagen - 79254	
QIAshredder	Qiagen - 79656	
PrimeScript RT Master Mix	Takara Biosciences - RR036B	
MouseWG-6 2.0	Illumina	
Bioanalyzer High Sensitivity DNA Analysis Kit	Agilent Technologies - 5067-4626	
Tissue Protein Extraction Reagent	Pierce Biotechnology - 78510	
Roche Complete Protease Inhibitor	Millipore Sigma - 11836153001	
Bradford Protein Assay Kit I	Bio-Rad - 5000001	
Protein Assay Standard I	Bio-Rad - 5000005	
Immun-Star™ WesternC™ Chemiluminescence Kit	Bio-Rad - 170-5070	
Paraformaldehyde, 4% w/v aq. soln.	TFS - 047392.9M	
Antigen Retrieval Buffer	Abcam - ab93678	
ProLong Diamond Anti-Fade Mounting Media with DAPI	TFS - P36962	RRID:AB_2307445
Click-iT EdU Cell Proliferation Kit for Imaging, AF 488 dye	TFS- C10337	

**Small Intestine Organoid Media**		
R Spondin-1 Conditioned Media (10mL)	HDDC Organoid Core	
Advanced DMEM/F-12 (85mL)	Life Technologies - 12634028	
Glutamax (100X) (1mL)	Life Technologies - 35050061	
HEPES (1M)(1mL)	Life Technologies - 15630080	
Primocin (50mg/mL) (200µL)	Invivogen - ant-pm-1	
Normocin (50mg/mL) (200µL)	Invivogen - ant-nr-1	
B27 Supplement (50X) (1mL)	Life Technologies 12587010	
N_2 Supplement (100X) (500µL)	Life Technologies 17502001	
N-Acetyl-Cysteine (500mM) (250µL)	Sigma - A7250	
Noggin (100ug/mL) (100µL)	Peprotech - 250-38	
EGF(500ug/mL) (10µL)	Peprotech - 315-09	
**Antibodies**		
Ghost Dye Violet 510	TonboBio - 13-0870-T100	
Mouse BD Fc Block (CD16/32)	BD Biosciences - 553142	RRID:AB_394656
Recombinant Anti-Lysozyme	Abcam - Ab108508	RRID:AB_10861277
Chromogranin A Polyclonal Antibody	TFS - PA5-77917	RRID:AB_2735659
Goat anti-Rabbit IgG Secondary Antibody, AF 555	Invitrogen - A-21428	RRID:AB_2535849
Goat anti-rabbit secondary antibody	Cell Signaling - 7074	RRID:AB_2099233
Donkey anti-goat secondary antibody	SouthernBiotech - 6402	RRID:AB_2796335
Goat anti-Rat IgG - Alexa Fluor™ 555	Invitrogen - A-21434	RRID:AB_2535855
Chicken anti-Mouse IgG - AF™ 647	Invitrogen- A-21463	RRID:AB_2535869
Goat anti-Rabbit IgG - AF™ 555	Invitrogen- A-21428	RRID:AB_2535849
Goat anti-Hamster IgG (H+L) Cross-Adsorbed Secondary Antibody, Alexa Fluor™ 647	Invitrogen - A-21451	RRID:AB_2535868
**Primer Sequences**		
Bacterial 16S, V4-V5, 450 bp	515F	GTGCCAGCGCCGCGGTAA

	907R	CCGTCAATTCCTTTGAGTTT
16S primers for RT-qPCR	Forward	CAGCMGCCGCGGTAA
	Reverse	CCGTCAATTCCTTTGAGTTT
TaqMan Gene Expression Assay: *Lyz1*	Invitrogen - 4331182	Mm00657323_m1
Defa20, Defa2, Gm21002	Invitrogen - 4331182	Mm00842045_91
Pnliprp2	Invitrogen - 4331182	Mm00448214_m1
Dmbt1	Invitrogen - 4331182	Mm00455996_m1
**Organisms**		
C57Bl/6J mouse	Jackson Laboratory - 000664	RRID:IMSR_JAX:000664
C57BL/6NCrl mouse	Charles River - 027	RRID:IMSR_CRL:027
**Equipment**		
ProOx 360 controller	BioSpherix	RRID:SCR_021130
A1R HD confocal microscope	Nikon	RRID:SCR_020317
flexiVent	SCIREQ	RRID:SCR_022673
2100 Bioanalyzer Instrument	Agilent	RRID:SCR_018043
QuantStudio 6	Applied Biosciences	RRID:SCR_020239
Rotor-Gene Q Series	Qiagen	RRID:SCR_018976
NovoSeq 6000 system	Illumina	RRID:SCR_016387
MiSeq platform	Illumina	RRID:SCR_020133
HiSeq 4000 platform	Illumina	RRID:SCR_016386
**Software**		
FACSDiva	BD Biosciences - v9.0	RRID:SCR_001456
FlowJo for Mac	BD Biosciences - v10.8.1	RRID:SCR_008520
Rotor-Gene Q Series Software	Qiagen	RRID:SCR_015740
FIJI	https://fiji.sc	RRID:SCR_002285
Prism 9 for Mac	GraphPad - v9.4.1	RRID:SCR_002798
R	R project for statistical computing - v4.2.2	RRID:SCR_001905
RStudio	Posit - v2022.12.0+353	RRID:SCR_000432

Vegan, R Package	https://github.com/vegandevs/vegan - v2.6-4	RRID:SCR_011950
phyloseq, R Package	https://github.com/joey711/phyloseq - v1.42.0	RRID:SCR_013080
DESeq2, R Package	10.18129/B9.bioc.DESeq2 - v1.38.2	RRID:SCR_015687
MaAsLin2, R Package	https://github.com/biobakery/Maaslin2 - v1.12.0	RRID:SCR_023241
tidyverse, R Package	https://www.tidyverse.org/ - v1.3.2	RRID:SCR_019186
caret, R Package	https://github.com/topepo/caret/ - v6.0-93	RRID:SCR_021138
SpiecEasi, R Package	https://github.com/zdk123/SpiecEasi - v1.1.2	RRID:SCR_022712
QIIME 2	QIIME 2 Core 2022.11	RRID:SCR_008249
SILVA	SILVA v138.1	RRID:SCR_006423
CLC Genomic Workbench	Qiagen - v22	RRID:SCR_011853
ClustVis	https://biit.cs.ut.ee/clustvis/	RRID:SCR_017133
Ingenuity Pathway Analysis	Qiagen (July 2022 edition)	RRID:SCR_008653
**Datasets**		
Gene-expression array, mouse	This paper	NCBI SRA: PRJNA931604
RNAseq, mouse	This paper	NCBI SRA: PRJNA931604
RNAseq, mouse organoid	This paper	NCBI SRA: PRJNA931604
16S rRNA MiSeq, mouse	This paper	NCBI SRA: PRJNA931604

Lead Contact: Further information and requests for resources and reagents should be directed to and will be fulfilled by the lead contact, Dr. Kent Willis (kawillis@uab.edu).

Materials Availability: This study did not generate any unique reagents or transgenic animals.

Data Availability:

- 16S and RNA sequencing data were deposited at the NCBI Sequence Read Archive (BioProject ID: PRJNA931604).
- Gene expression array data were deposited at the NCBI Gene Expression Omnibus (Accession number: GSE125489).
- Processed data files are available at http://github.com/WillisLungLab/AMP.
- This manuscript does not report original code.
- All other data needed to evaluate the conclusions in the manuscript are available within the main text or supplementary materials.

Murine Small Intestine (ENR) Media (100mL)

**Component**	**Volume**	**Catalog #**	**Final Concentration**	**Purpose**
Rspondin-1 Conditioned Media	10mL	HDDC Organoid Core	10%	Contributes Rspondin-1 for stem cell proliferation
Advanced DMEM/F-12	85mL	Life Technologies 12634028	85%	Contributes glucose, NEAAs, sodium pyruvate, phenol red
Glutamax (100X)	1mL	Life Technologies 35050061	1X	Contributes L-glutamine to support growth
HEPES (1M)	1mL	Life Technologies 15630080	10mM	pH buffer for CO_2 changes
Primocin (50mg/mL)	200uL	Invivogen ant-pm-1	100ug/mL	Antibiotic
Normocin (50mg/mL)	200uL	Invivogen ant-nr-1	100ug/mL	Antibiotic
B27 Supplement (50X)	1mL	Life Technologies 12587010	0.5X	Growth supplement

N2 Supplement (100X)	500uL	Life Technologies 17502001	0.5X	Growth supplement
N-Acetyl-Cysteine (500mM)	250uL	Sigma A7250	1.25mM	Antioxidant
Noggin (100ug/mL)	100uL	Peprotech 250-38	100ng/mL	BMP inhibitor
EGF(500ug/mL)	10uL	Peprotech 315-09	50ng/mL	Epidermal growth factor, stimulates mitosis

Supplemental Information

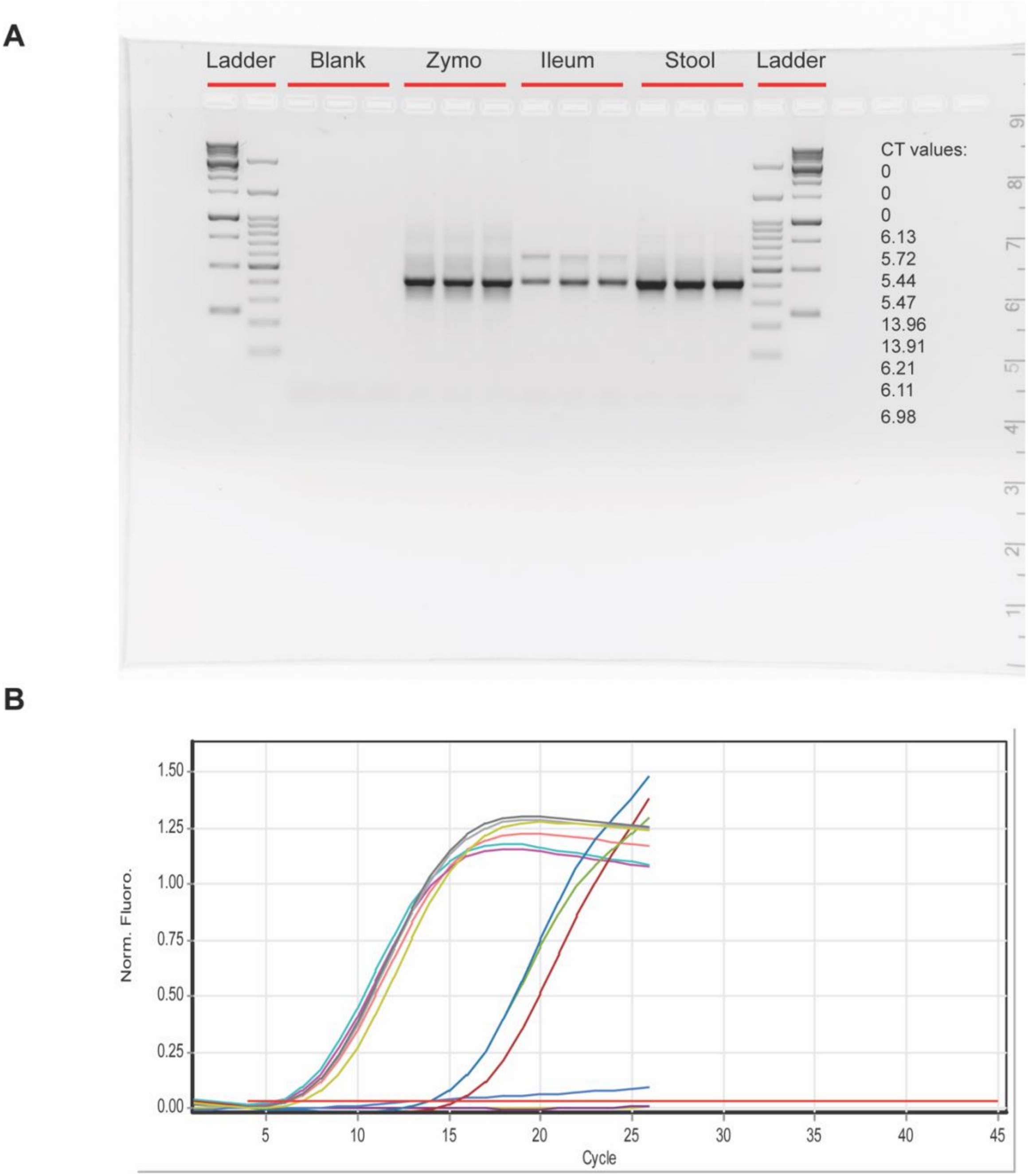

Figure S1. Ileal intestinal colonization is a lower biomass than stool by RT-qPCR.

A) Gel showing relative abundance of 16s rRNA gene copies per ug in triplicate blank samples, ZymoBIOMICS organism positive controls, ileal samples, and stool samples. (CT values corresponding to each band are shown on the left of the gel.

B) Cycle threshold graph for the selected RT-qPCR samples above.

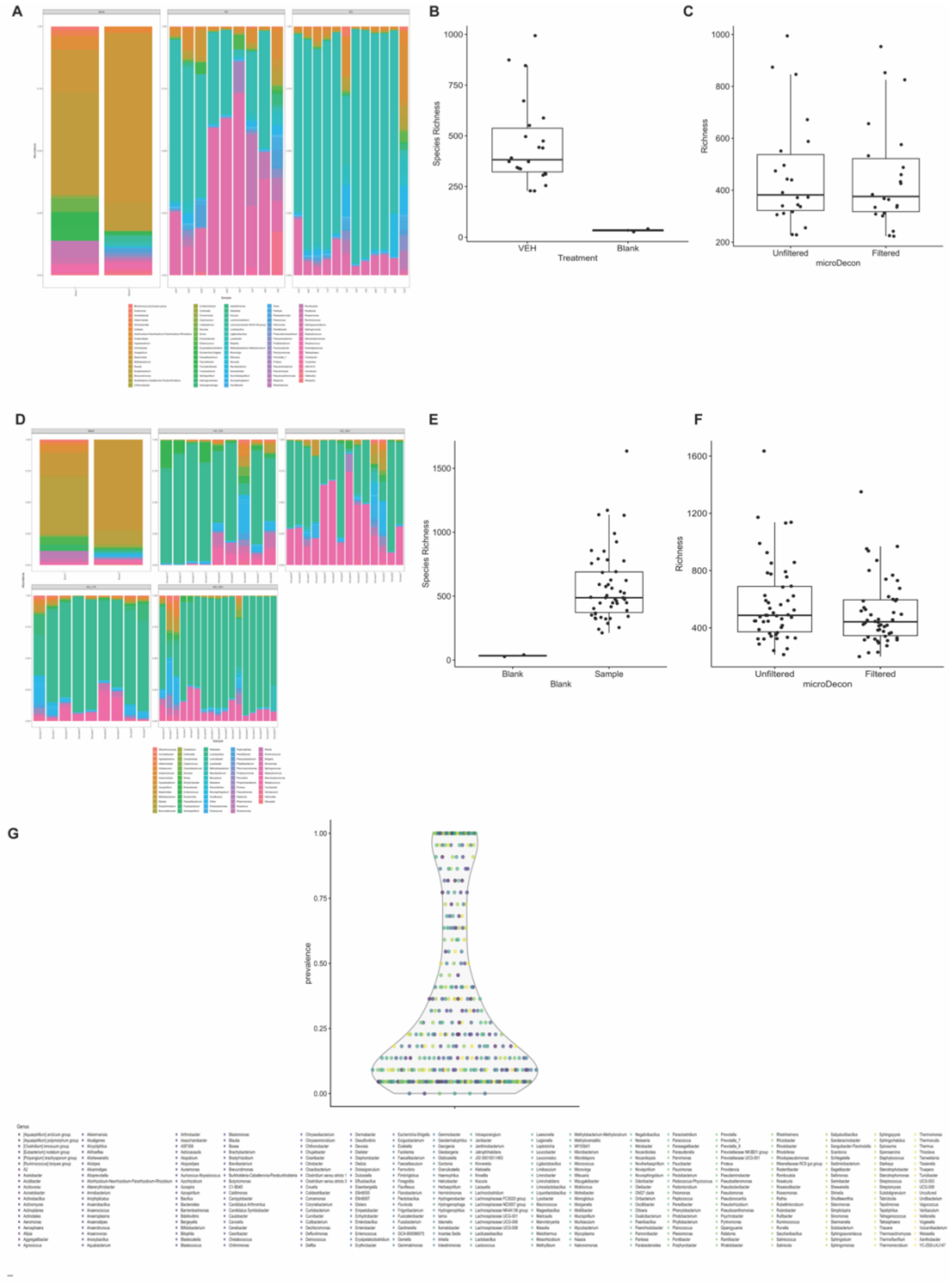

Figure S2. Quality control figure showing sequencing contaminates removed by microDecon.

A) Relative abundance of negative sequencing controls (blanks) versus samples in experiment 1.

B) Species richness of blanks versus samples.

C) Species richness before and after performing decontamination.

D) Relative abundance of negative sequencing controls (blanks) versus samples in experiment 2.
E) Species richness of blanks versus samples.
F) Species richness before and after performing decontamination.
G) Prevalence plot.

See output plots for microDecon at github.com/WillisLungLab/AMP.

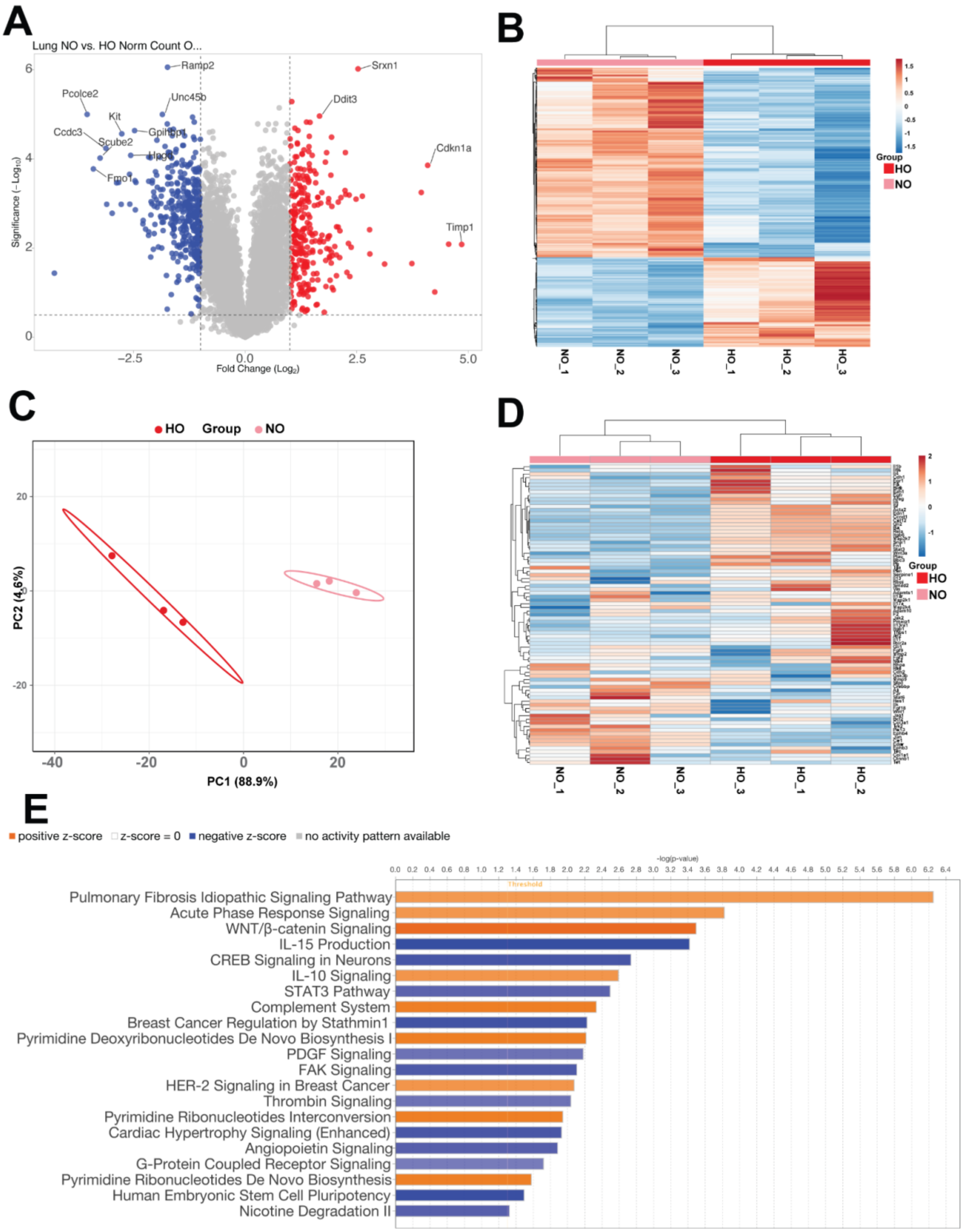
A
Lung NO vs. HO Norm Count O...
Ramp2
Srxn1
Pcolce2
Unc45b
Ddit3
Kit
Ccdc3
Scube2
Cdkn1a
Fmo1
Timp1
Significance (−Log10)
Fold Change (Log2)
−2.5
0.0
2.5
5.0
B
Group
HO
NO
NO_1
NO_2
NO_3
HO_1
HO_2
HO_3
C
HO
Group
NO
PC2 (4.6%)
PC1 (88.9%)
D
Group
HO
NO
NO_1
NO_2
NO_3
HO_3
HO_1
HO_2
E
positive z-score
z-score = 0
negative z-score
no activity pattern available
-log(p-value)
Threshold
Pulmonary Fibrosis Idiopathic Signaling Pathway
Acute Phase Response Signaling
WNT/β-catenin Signaling
IL-15 Production
CREB Signaling in Neurons
IL-10 Signaling
STAT3 Pathway
Complement System
Breast Cancer Regulation by Stathmin1
Pyrimidine Deoxyribonucleotides De Novo Biosynthesis I
PDGF Signaling
FAK Signaling
HER-2 Signaling in Breast Cancer
Thrombin Signaling
Pyrimidine Ribonucleotides Interconversion
Cardiac Hypertrophy Signaling (Enhanced)
Angiopoietin Signaling
G-Protein Coupled Receptor Signaling
Pyrimidine Ribonucleotides De Novo Biosynthesis
Human Embryonic Stem Cell Pluripotency
Nicotine Degradation II

Figure S3. Hyperoxia exposure alters the mouse lung transcriptome.

A) Volcano plot of lung gene expression array showing gene expression altered by hyperoxia exposure. NO, normoxia (pink). HO, hyperoxia (red).

B) Heatmap showing differential gene expression in normoxia versus hyperoxia-exposed mice.

C) Principal components analysis showing differential clustering of mice exposed to normoxia versus hyperoxia. PC, principal component.

D) Heatmap showing differential expression of pulmonary fibrosis-related genes.

E) Differentially expressed pathways in normoxia versus hyperoxia exposure, showing marked increases in fibrosis, acute phase response and WNT/β-catenin-related pathways.

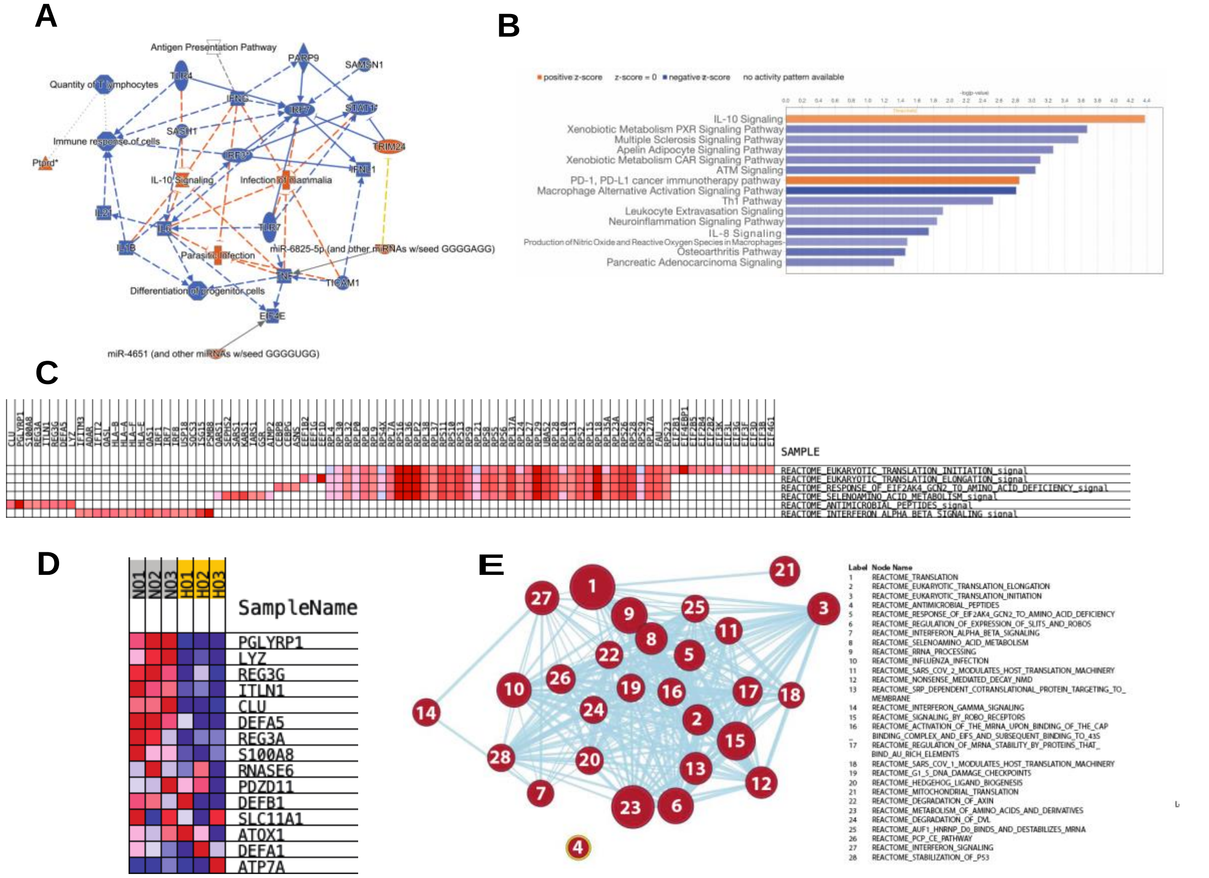

Figure S4. Hyperoxia exposure alters the mouse ileal transcriptome. Pathway analysis and GSEA corresponding to Figure 1 D-G.

A) Graphical summary of pathways and networks generated by Ingenuity Pathway Analysis (IPA).

B) Top canonical pathways (IPA).

C) Heatmap of normalized enrichment scores (NES) of genes in top gene lists
D) Heatmap showing expression of key antimicrobial peptides in hyperoxia (HO) versus normoxia (NO) corresponding to GSEA.
E) GSE Map of gene sets with FDR (q) values <0.05 and Jaccard coefficients <0.01

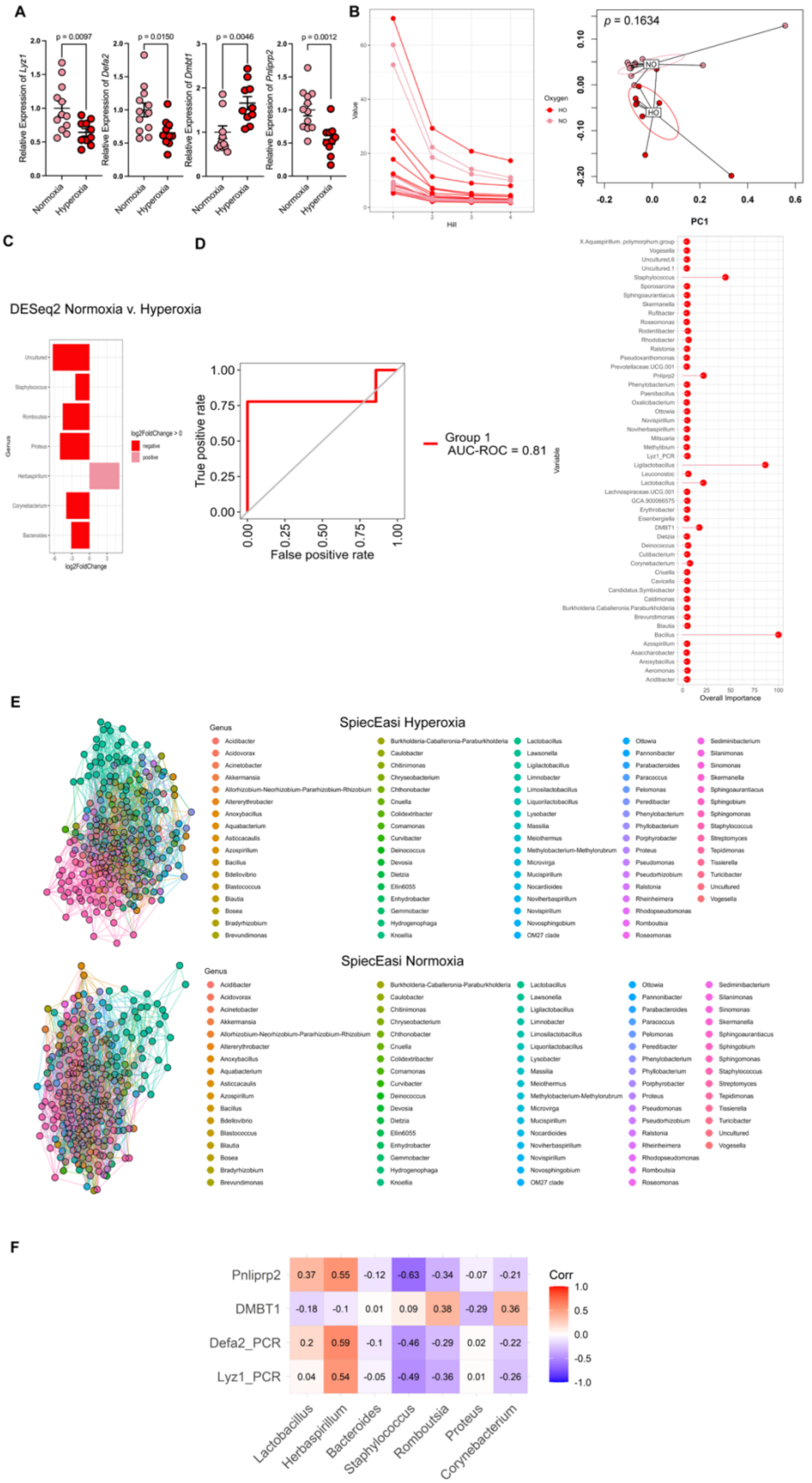

A
p = 0.0097
p = 0.0150
p = 0.0046
p = 0.0012
Relative Expression of Lyz1
Relative Expression of Defa2
Relative Expression of Dmbt1
Relative Expression of Pnliprp2
Normoxia
Hyperoxia
B
Value
Hill
Oxygen
HO
NO
p = 0.1634
NO
HO
PC1
C
DESeq2 Normoxia v. Hyperoxia
Uncultured
Staphylococcus
Romboutsia
Proteus
Herbaspirillum
Corynebacterium
Bacteroides
Genus
log2FoldChange
log2FoldChange > 0
negative
positive
D
True positive rate
False positive rate
Group 1
AUC-ROC = 0.81
Variable
Overall Importance
E
SpiecEasi Hyperoxia
Genus
SpiecEasi Normoxia
F
Pnliprp2
DMBT1
Defa2_PCR
Lyz1_PCR
0.37
0.55
-0.12
-0.63
-0.34
-0.07
-0.21
-0.18
-0.1
0.01
0.09
0.38
-0.29
0.36
0.2
0.59
-0.1
-0.46
-0.29
0.02
-0.22
0.04
0.54
-0.05
-0.49
-0.36
0.01
-0.26
Corr
Lactobacillus
Herbaspirillum
Bacteroides
Staphylococcus
Romboutsia
Proteus
Corynebacterium

Figure S5. Hyperoxia alters the ileal microbiome.

A) Quantitative PCR for a selection of differentially expressed genes showing alterations in expression of antimicrobial peptides and Paneth cell markers.
B) Renyi-Hill diagram.
C) Principal components analysis of Weighted UniFrac, p = 0.1634, pseudo-F = 1.8708 PERMANVOA, p = 0.7553 PERMDISP.
D) Binomial regression using DESeq2 showing differences at the genera level in normoxia- or hyperoxia-exposed animals.
E) Random forest model with important contributions from the genera *Staphylococcus*, *Ligilactobacilus*, and *Bacillus*.
F) SpecEasi network analysis of the microbiota of hyperoxia and normoxia exposed mice.
G) Relative abundance at the genera level.
H) Spearman correlation analysis showing association between antimicrobial peptide expression by qPCR and differentially abundant genera. Correlation coefficients are displayed.

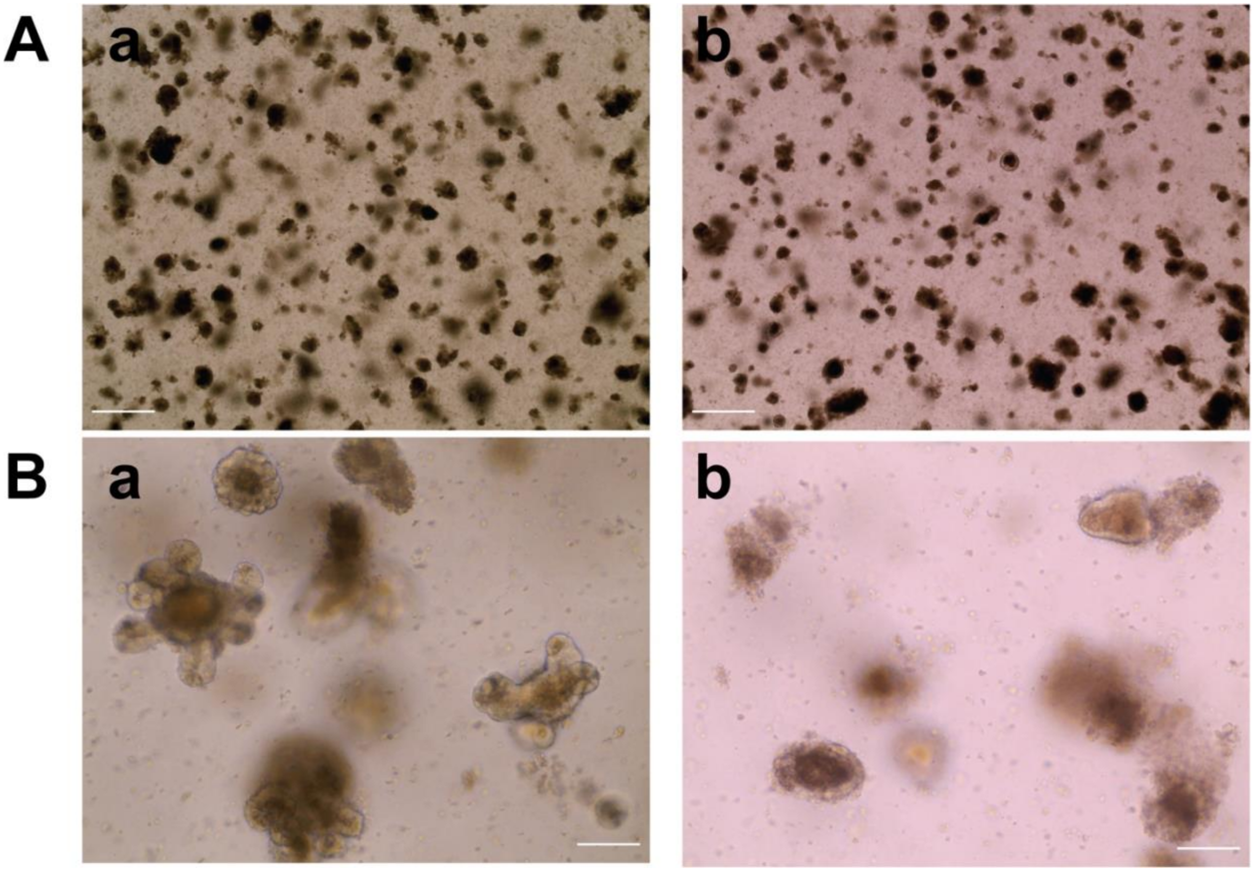

Figure S6. Hyperoxia exposure alters the morphology of intestinal organoids.

A) Visual light microscopy at 4x of intestinal organoids after exposure to (a) normoxia or (b) hyperoxia for 24 h. Scale bar represents 1000 μm.

B) Visual light microscopy and 20x magnification showing less budding and differences in epithelial integrity in (b) hyperoxia exposed organoids. Scale bar represents 200 μm.

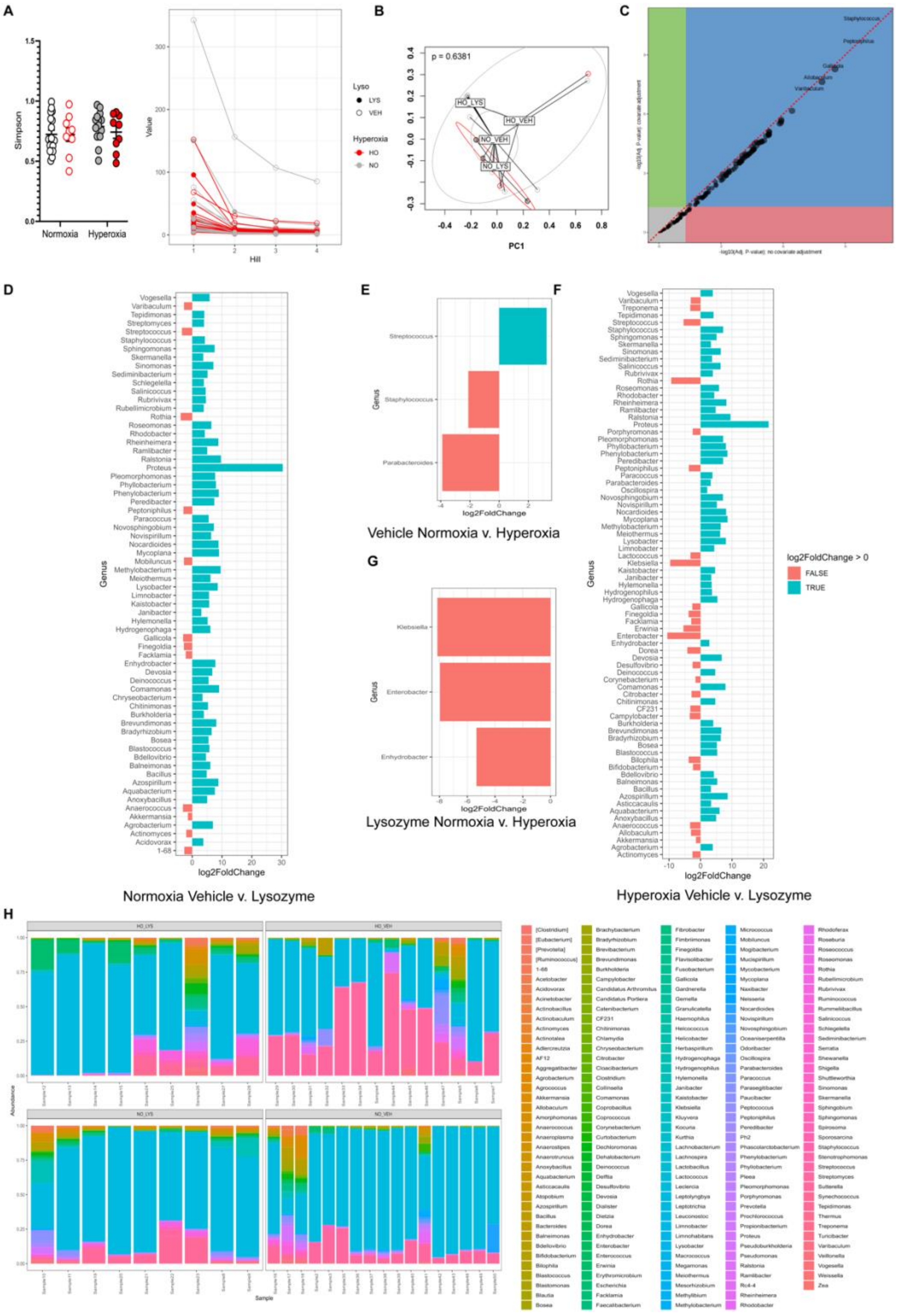

Figure S7. Lysozyme and hyperoxia alter the ileal microbiome.

A) Alpha diversity quantified by the Simpson index and Renyi-Hill number diversity.

B) Principal components analysis of weighted UniFrac p = 0.6381, pseudo-F = 0.6680 PERMANOVA, p = 0.3445 PERMDISP.

C) MaAsLin 2 (Microbiome Multivariable Associations with Linear Models) showing differences in the intestinal microbiota.
D) Binomial regression showing differences at the genera level in normoxia between vehicle and lysozyme exposed animals.
E) Binomial regression showing differences between normoxia and hyperoxia vehicle-exposed control animals.
F) Binomial regression showing differences between normoxia and hyperoxia lysozyme-exposed animals.
G) Binomial regression showing differences at the genera level in hyperoxia between vehicle- and lysozyme exposed animals.
H) Relative abundance at the genera level.

Table S1. Paneth cell markers significantly changed in the murine ileum in response to hyperoxia.

Symbol	Fold Change	*P*	Name
Pnliprp2	-5.01959	0.004608	pancreatic lipase-related protein 2
Lyz1	-4.10972	0.012384	lysozyme 1
Dmbt1	-3.8282	0.001136	Deleted In Malignant Brain Tumors 1
Gpx2	-3.32466	0.006985	glutathione peroxidase 2
Reg3g	-2.72808	0.008283	regenerating islet-derived 3 gamma
Guca2b	-2.43296	0.002597	guanylate cyclase activator 2b
Ang4	-2.37332	0.023716	angiogenin
Itln1	-2.10236	0.012832	intelectin 1
Defa1	-1.30565	0.038754	defensin, alpha 1

Table S2. Paneth cell markers significantly changed in the murine intestinal organoids in response to hyperoxia.

Symbol	Fold Change	*P*	Name
Mptx2	-6.10435	2.47E-06	mucosal pentraxin 2
Dmbt1	-5.79307	0.027013	deleted in malignant brain tumors 1
Defa26	-3.80301	1.77E-06	defensin, alpha, 26
Kcnn4	-2.89266	3.81E-05	potassium intermediate/small conductance calcium-activated channel, subfamily N, member 4
Defa23	-2.60226	0.000873	defensin, alpha, 23
Ang4	-2.38428	0.003666	angiogenin, ribonuclease A family, member 4
Defa20	-2.34975	0.013918	defensin, alpha, 20
Defa22	-2.2797	0.023124	defensin, alpha, 22
Defa21	-2.26413	0.027105	defensin, alpha, 21
Defa5	-2.20591	0.029043	defensin, alpha, 5
Wnt3	-1.70433	0.049799	wingless-type MMTV integration site family, member 3
Spink4	-1.65614	0.043295	serine peptidase inhibitor, Kazal type 4
Pnliprp2	-1.63384	0.000914	pancreatic lipase-related protein 2
Dll4	-1.54349	0.040336	delta like canonical Notch ligand 4
Rap1a	1.351647	0.054885	RAS-related protein 1a
Tm4sf20	1.902737	0.001682	transmembrane 4 L six family member 20
Clca1	2.660606	0.000193	chloride channel accessory 1
Guca2b	3.273845	1.09E-06	guanylate cyclase activator 2b (retina)
Guca2a	3.743392	6.98E-09	guanylate cyclase activator 2a (guanylin)

Table S3. Lung inflammation-related genes as compared to normoxia vehicle exposed mice.

	HO VEH vs. NO VEH		HO LYZ vs NO VEH		
Symbol	Fold Change	q	Fold Change	q	Database object name
Ahsg	626.6391	0.005346	21.07616	0.269682	alpha-2-HS-glycoprotein
Csrp3	14.15701	0.000613	-1.133	0.927459	cysteine and glycine-rich protein 3
Mylk3	6.296584	0.000862	-1.28659	0.76882	myosin light chain kinase 3
Il1r2	3.475312	0.025753	1.949132	0.294827	interleukin 1 receptor, type II
Ptger3	2.537922	0.000685	1.6335	0.116541	prostaglandin E receptor 3 (subtype EP3)
Pbk	2.51287	0.017812	1.556554	0.330692	PDZ binding kinase
Ccl6	2.087173	4.42E-06	1.087488	0.740466	chemokine (C-C motif) ligand 6
Clec7a	1.904633	4.27E-06	-1.19229	0.341095	C-type lectin domain family 7, member a
Gpx1	1.797218	2.05E-06	1.19287	0.262587	glutathione peroxidase 1
Ccr2	1.754312	0.026097	1.488087	0.138412	chemokine (C-C motif) receptor 2
Irf5	1.608403	0.014589	1.329064	0.186512	interferon regulatory factor 5
Chid1	1.593086	0.006607	1.357239	0.100582	chitinase domain containing 1
Ndufs4	1.580271	0.000592	1.035841	0.872654	NADH:ubiquinone oxidoreductase core subunit S4
Ctla2a	1.517331	0.00679	1.172581	0.402791	cytotoxic T lymphocyte-associated protein 2 alpha
Stk39	1.513097	0.043299	1.442597	0.074579	serine/threonine kinase 39
Bst1	1.51008	0.035102	1.110652	0.693385	bone marrow stromal cell antigen 1
Dicer1	-1.58638	0.000146	-1.1519	0.361353	dicer 1, ribonuclease type III
F2r	-1.67139	3.58E-05	-1.13661	0.444461	coagulation factor II (thrombin) receptor
Ackr2	-1.69257	0.00167	-1.08879	0.730939	atypical chemokine receptor 2
Kdm6b	-1.6956	2.7E-05	-1.29584	0.071318	KDM1 lysine (K)-specific demethylase 6B
Akna	-1.73878	0.001889	-1.44747	0.052764	AT-hook transcription factor
Cybb	-1.75339	0.001212	-1.36063	0.113292	cytochrome b-245, beta polypeptide
Cdh5	-1.78237	2.99E-05	-1.12635	0.542984	cadherin 5
Adora2a	-2.30738	0.000152	-1.4867	0.113343	adenosine A2a receptor
C1qtnf3	-2.98067	0.006724	-1.60992	0.30865	C1q and tumor necrosis factor related protein 3

Table S4. Genes-related to endothelial cell regulation.

	HO VEH vs. RA VEH		HO LYZ vs RA VEH		
Symbol	FC	q	FC	q	Database object name
Mt3	171.49	0.039911	79.74	0.085312	metallothionein 3
Gata4	3.57	0.001527	1.45	0.48924	GATA binding protein 4
Igf2	2.56	8.07E-05	-1.22	0.560584	insulin-like growth factor 2
Aldh1a2	2.41	2.33E-05	1.19	0.560409	aldehyde dehydrogenase family 1, subfamily A2
Smoc2	2.10	1.53E-05	1.08	0.785722	SPARC related modular calcium binding 2
Gpx1	1.80	2.05E-06	1.19	0.262587	glutathione peroxidase 1
Ccr2	1.75	0.026097	1.49	0.138412	chemokine (C-C motif) receptor 2
Itgb1bp1	1.69	0.000516	1.25	0.216524	integrin beta 1 binding protein 1
Nr4a1	1.67	0.034821	-1.17	0.62015	nuclear receptor subfamily 4, group A, member 1
Mydgf	1.60	0.000569	1.32	0.060535	myeloid derived growth factor
Fgf18	1.50	0.013835	1.03	0.916387	fibroblast growth factor 18
Stard13	-1.50	0.025186	-1.04	0.9001	StAR-related lipid transfer (START) domain containing 13
Jup	-1.50	0.003187	-1.18	0.324177	junction plakoglobin
Cxcl12	-1.50	0.01231	1.01	0.985026	chemokine (C-X-C motif) ligand 12
Paxip1	-1.55	0.005012	-1.24	0.22634	PAX interacting (with transcription-activation domain) protein 1
Crkl	-1.56	0.003138	-1.03	0.909193	v-crk avian sarcoma virus CT10 oncogene homolog-like
Dicer1	-1.59	0.000146	-1.15	0.361353	dicer 1, ribonuclease type III
Mecp2	-1.59	0.000473	-1.08	0.682247	methyl CpG binding protein 2
Jag1	-1.59	0.002791	1.14	0.53448	jagged 1
Flt1	-1.59	0.008216	1.03	0.929469	FMS-like tyrosine kinase 1
Ece1	-1.64	1.62E-05	-1.19	0.207628	endothelin converting enzyme 1
Fzd4	-1.67	0.034973	1.25	0.431742	frizzled class receptor 4
Atoh8	-1.68	0.001662	-1.39	0.06783	atonal bHLH transcription factor 8
Kdm6b	-1.70	2.7E-05	-1.30	0.071318	KDM1 lysine (K)-specific demethylase 6B
Ptprm	-1.71	2.19E-05	-1.06	0.778646	protein tyrosine phosphatase, receptor type, M
Sp1	-1.72	4.56E-05	-1.20	0.26853	trans-acting transcription factor 1
Cdh5	-1.78	2.99E-05	-1.13	0.542984	cadherin 5
Pecam1	-1.79	2.22E-05	-1.23	0.220446	platelet/endothelial cell adhesion molecule 1
Pde4d	-1.81	0.000142	-1.33	0.104711	phosphodiesterase 4D, cAMP specific
Flt4	-1.87	1.53E-06	-1.18	0.332498	FMS-like tyrosine kinase 4
Il6ra	-2.55	4E-05	-1.56	0.084073	interleukin 6 receptor, alpha
Lgals12	-5.58	0.043358	1.26	0.832064	lectin, galactose binding, soluble 12

Fgf2	-6.15	0.047026	2.32	0.342984	fibroblast growth factor 2

Table S5. Genes with opposite regulation associated with lysozyme supplementation.

	HO_VEH vs. RA_VEH FC	HO_VEH vs. RA_VEH q	HO_LYZ vs. RA_VEH FC	HO_LYZ vs. RA_VEH q	HO_LYZ vs. HO_VEH FC	HO_LYZ vs. HO_VEH q	Database object name
Sln	13.69	0.000426	-11.55	0.003672	-158.08	2.94E-10	sarcolipin
Myl7	6.38	0.001178	-4.37	0.013565	-27.89	4.11E-09	myosin, light polypeptide 7, regulatory
Hba-a2	4.21	0.000184	-2.45	0.032445	-10.30	1.53E-09	hemoglobin alpha, adult chain 2
Hbb-bt	4.16	0.000119	-2.83	0.007483	-11.79	2.44E-11	hemoglobin, beta adult t chain
Hba-a1	3.87	0.000579	-2.65	0.019222	-10.22	2.74E-09	hemoglobin alpha, adult chain 1
Snca	3.63	0.000852	-2.38	0.038694	-8.65	2.51E-08	synuclein, alpha
Alas2	3.61	0.000565	-3.21	0.001881	-11.59	2.27E-11	aminolevulinic acid synthase 2, erythroid
Hbb-bs	2.97	0.016819	-4.08	0.000942	-12.09	4.03E-09	
Slc4a1	2.47	0.008169	-2.32	0.015472	-5.74	1.53E-07	solute carrier family 4 (anion exchanger), member 1
Cpe	1.74	0.000235	-1.75	0.000201	-3.03	5.23E-14	carboxypeptidase E
Igfbp5	1.51	0.041052	-1.82	0.001169	-2.75	4.24E-08	insulin-like growth factor binding protein 5
mt-Rnr2	1.51	0.016924	-1.60	0.004611	-2.41	5.45E-08	mitochondrially encoded 16S rRNA
S100a1	1.39	0.036962	-1.41	0.023687	-1.97	7.7E-06	S100 calcium binding protein A1
Glg1	-1.53	0.034846	1.86	0.000484	2.84	6.73E-09	golgi apparatus protein 1
Abhd2	-1.99	0.003723	1.79	0.014075	3.56	3.21E-08	abhydrolase domain containing 2

Table S5. Enrichment analysis table.

**Node Name**	**FDR (q)**	**Gene Set Size**	**NES**
REACTOME_TRANSLATION	<1E-04	231	2.4031

REACTOME_EUKARYOTIC_TRANSLATION_ELONGATION	<1E-04	78	2.3969
REACTOME_EUKARYOTIC_TRANSLATION_INITIATION	<1E-04	98	2.3784
REACTOME_ANTIMICROBIAL_PEPTIDES	3.00E-04	15	2.3316
REACTOME_RESPONSE_OF_EIF2AK4_GCN2_TO_AMINO_ACID_DEFICIENCY	4.00E-04	82	2.2736
REACTOME_REGULATION_OF_EXPRESSION_OF_SLITS_AND_ROBOS	5.00E-04	134	2.2938
REACTOME_INTERFERON_ALPHA_BETA_SIGNALING	9.00E-04	38	2.2267
REACTOME_SELENOAMINO_ACID_METABOLISM	0.001	93	2.2335
REACTOME_RRNA_PROCESSING	0.001	136	2.2388
REACTOME_INFLUENZA_INFECTION	0.0011	120	2.1934
REACTOME_SARS_COV_2_MODULATES_HOST_TRANSLATION_MACHINERY	0.0012	40	2.1739
REACTOME_NONSENSE_MEDIATED_DECAY_NMD	0.0014	91	2.1568
REACTOME_SRP_DEPENDENT_COTRANSLATIONAL_PROTEIN_TARGETING_TO_MEMBRANE	0.0025	95	2.1232
REACTOME_INTERFERON_GAMMA_SIGNALING	0.0029	47	2.1057
REACTOME_SIGNALING_BY_ROBO_RECEPTORS	0.0041	158	2.0409
REACTOME_ACTIVATION_OF_THE_MRNA_UPON_BINDING_OF_THE_CAP_BINDING_COMPLEX_AND_EIFS_AND_SUBSEQUENT_BINDING_TO_43S	0.0041	49	2.0739
REACTOME_REGULATION_OF_MRNA_STABILITY_BY_PROTEINS_THAT_BIND_AU_RICH_ELEMENTS	0.0042	66	2.0695
REACTOME_SARS_COV_1_MODULATES_HOST_TRANSLATION_MACHINERY	0.0042	31	2.0266
REACTOME_G1_S_DNA_DAMAGE_CHECKPOINTS	0.0042	51	2.0493
REACTOME_HEDGEHOG_LIGAND_BIOGENESIS	0.0043	49	2.0314
REACTOME_MITOCHONDRIAL_TRANSLATION	0.0044	74	1.9974
REACTOME_DEGRADATION_OF_AXIN	0.0045	46	2.0154
REACTOME_METABOLISM_OF_AMINO_ACIDS_AND_DERIVATIVES	0.0046	207	2.0027

REACTOME_DEGRADATION_OF_DVL	0.0046	44	2.0005
REACTOME_AUF1_HNRNP_D0_BINDS_AND_DESTABILIZES_MRNA	0.0046	45	2.012
REACTOME_PCP_CE_PATHWAY	0.0047	64	1.9909
REACTOME_INTERFERON_SIGNALING	0.0047	112	2.0068
REACTOME_STABILIZATION_OF_P53	0.005	47	1.9831

References

1. Kilkenny, C., Browne, W.J., Cuthill, I.C., Emerson, M., and Altman, D.G. (20*10*). Improving bioscience research reporting: the ARRIVE guidelines for reporting animal research.
2. Willis, K.A., Siefker, D.T., Aziz, M.M., White, C.T., Mussarat, N., Gomes, C.K., Bajwa, A., Pierre, J.F., Cormier, S.A., and Talati, A.J. (201*9*). Perinatal maternal antibiotic exposure augments lung injury in offspring in experimental bronchopulmonary dysplasia. American Journal of Physiology - Lung Cellular and Molecular Physiology *3*, 21. 10.1152/ajplung.00561.2018.
3. Nardiello, C., Mižíková, I., Silva, D.M., Ruiz-Camp, J., Mayer, K., Vadász, I., Herold, S., Seeger, W., and Morty, R.E. (2017). Standardisation of oxygen exposure in the development of mouse models for bronchopulmonary dysplasia. Disease models & mechanisms 10, 185–196. 10.1242/dmm.027086.
4. Wang, W., Yan, T., Guo, W., Niu, J., Zhao, Z., Sun, K., Zhang, H., Yu, Y., and Ren, T. (2021). Constitutive GLI1 expression in chondrosarcoma is regulated by major vault protein via mTOR/S6K1 signaling cascade. Cell Death Differ *28*, 2221–2237. 10.1038/s41418-021-00749-4.
5. Metsalu, T., and Vilo, J. (2015). ClustVis: a web tool for visualizing clustering of multivariate data using Principal Component Analysis and heatmap. Nucleic Acids Res *43*, W566–W570. 10.1093/nar/gkv468.
6. Baker, J.M., Hinkle, K.J., McDonald, R.A., Brown, C.A., Falkowski, N.R., Huffnagle, G.B., and Dickson, R.P. (2021). Whole lung tissue is the preferred sampling method for amplicon-based characterization of murine lung microbiota. Microbiome 9, 99. 10.1186/s40168-021-01055-4.
